# Challenges and Promises for Planning Future Clinical Research Into Bacteriophage Therapy Against *Pseudomonas aeruginosa* in Cystic Fibrosis. An Argumentative Review

**DOI:** 10.3389/fmicb.2018.00775

**Published:** 2018-05-04

**Authors:** Martina Rossitto, Ersilia V. Fiscarelli, Paola Rosati

**Affiliations:** ^1^Cystic Fibrosis Microbiology, Laboratory Department, Bambino Gesù Children's Hospital IRCCS, Rome, Italy; ^2^Unit of Clinical Epidemiology, Bambino Gesù Children's Hospital IRCCS, Rome, Italy

**Keywords:** bacteriophage therapy, efficiency of plating, lytic phages, phage cocktails, phages and antibiotics combined, multidrug-resistant *Pseudomonas aeruginosa*, biofilm, cystic fibrosis

## Abstract

Although early aggressive and prolonged treatment with specific antibiotics can extend survival in patients with cystic fibrosis (CF) colonized by opportunistic *Pseudomonas aeruginosa* (PA), antibiotics fail to eradicate the infecting multidrug-resistant (MDR) PA strains in CF. Century-long research has suggested treating patients with bacteriophages (phages, prokaryotic viruses) naturally hosted by bacteria. Although the only phage types used in therapy, lytic phages, lyse PA aggregated in biofilm matrix by depolymerase degrading enzymes, how they can effectively, safely, and persistently do so in patients with CF is unclear. Even though advanced techniques for formulating phage cocktails, training phages and collecting phage libraries have improved efficacy *in vitro*, whether personalized or ready-to-use therapeutic approaches or phages and antibiotics combined are effective and safe *in vivo*, and can reduce PA biofilms, remains debatable. Hence, to advance clinical research on phage therapy in clinical trials, also involving mucoid and non-mucoid multidrug-resistant PA in CF, and overcome problems in Western international regulations, we need reliable and repeatable information from experiments *in vitro* and *in vivo* on phage characterization, cocktail selection, personalized approaches, and phages combined with antibiotics. These findings, challenges, and promises prompted us to undertake this argumentative review to seek up-to-date information from papers describing lytic phage activity tested *in vitro* on PA laboratory strains, and PA strains from chronic infections including CF. We also reviewed *in vivo* studies on phage activity on pulmonary and non-pulmonary animal host models infected by laboratory or CF PA strains. Our argumentative review provides essential information showing that future phage clinical research in CF should use well-characterized and selected phages isolated against CF PA, tested *in vitro* under dynamic conditions in cocktails or combined with antibiotics, and *in vivo* on non-pulmonary and pulmonary host models infected with mucoid and non-mucoid CF MDR PA. Our findings should encourage pharmaceutical industries to conduct clinical trials *in vitro* and *in vivo* testing patented genomic engineered phages from phage libraries combined with antibiotics to treat or even prevent multidrug-resistant PA in CF, thus helping international regulatory agencies to plan future clinical research on phage therapy in CF.

## Introduction

*Pseudomonas aeruginosa* (PA), an opportunistic environmental pathogen, typically colonizes 30% of children, and up to 80% of 25-year-old and older adults with cystic fibrosis (CF) (Gibson et al., 2003; Stuart et al., 2010), thus inexorably causing chronic lung infection, pulmonary function decline, and death (Frederiksen et al., 1997; Murray et al., 2007; Moreau-Marquis et al., 2008). In CF lung, PA typically produces biofilm, a deleterious, complex tower- or mushroom-like matrix enclosing sessile PA aggregates. Surface motile planktonic PA cells leave the biofilm, colonize new lung sites, and initiate new sessile PA micro-colonies triggering repeated lung infections requiring antibiotics (Supplementary Table 1; Costerton et al., 1999, 2003). After prolonged and repeated broad-spectrum antibiotic courses, enhanced resistance to antibiotics develops and leads to non-mucoid and mucoid multidrug-resistant (MDR) PA strains (Donlan and Costerton, 2002; Li et al., 2005; Gómez and Prince, 2007; Rasamiravaka et al., 2015). MDR PA as defined by Centers for Disease Control and Prevention in Atlanta (Sievert et al., 2013) is non-susceptible (resistant or intermediate) to at least one agent in three or more antimicrobial classes: β-lactam, aminoglycosides, cephalosporins, fluoroquinolones, and carbapenems. Despite international recommendations on CF infections, including MDR organism prevention and control (Saiman et al., 2014), and recent findings on PA evolutionary adaptation, diversification, and resistance factors in CF lungs (Supplementary Table 1; Winstanley et al., 2016), research interest is waning on new antibiotics to include in pipeline programs (Theuretzbacher, 2009; Bassetti et al., 2011). Hence, to combat CF MDR PA infections, we urgently require more efficient therapeutic approaches. As a new weapon to treat CF lung infection caused by MDR PA and other multi-resistant superbugs, extensive research has reappraised bacteriophage (phage) therapy (Sulakvelidze et al., 2001; Thiel, 2004; Hurley et al., 2012).

Phages, bacterial parasitic viruses abounding in the environment, are the oldest known viruses dating back 3.5 billion years (Schopf, 1992; Ackermann, 1998). Although every bacterium probably hosts its own phages, they are deemed unable to produce infection in human organisms (Domingo-Calap et al., 2016). Phages were discovered independently about a century ago in stool samples taken from patients shortly before recovering from dysentery (Twort, 1915; D'Hérelle, 1917; Salmond and Fineran, 2015). These findings, therefore, suggested that phages might be useful for antibacterial therapy (Clokie et al., 2011; Reardon, 2014). Even though Eastern European health institutions officially permitted, and researchers clinically applied phage therapy in patients with CF (Shabalova et al., 1995; Kutateladze and Adamia, 2010; Krylov et al., 2016), in Western Europe international regulatory bodies were unable to solve ethical concerns or issue clinical guidelines on phage therapy in patients with CF (Verbeken et al., 2007). Hence, phage therapy gave way to the newly discovered antibiotics (Sulakvelidze et al., 2001; Salmond and Fineran, 2015). Unlike antibiotics, phages attack the host-pathogen surface, and inject their genome into the host cell leaving the commensal bacteria unchanged, thus gaining the nickname “intelligent antibiotics” (Loc-Carrillo and Abedon, 2011; Domingo-Calap et al., 2016). After injecting their genome, phages can have two different lifecycles. In the lytic cycle, the bacterial molecular system produces viral components such as capsid, nucleic acid, and structural proteins. Eventually, these assembled components release new virions thus causing bacterial lysis (Bradley and Robertson, 1968). In the lysogenic cycle, the phage lingers as a plasmid or integrates into the bacterial genome giving rise to a prophage. These quiescent states (plasmid or prophage) last until specific stimuli force them to enter the lytic cycle (Clokie et al., 2011; Salmond and Fineran, 2015). Because lysogenic (temperate) phages can transfer DNA host fragments possibly containing gene-encoding toxins or antibiotic resistance thus generating new virulent bacteria, the only phages exploited for therapy are lytic phages. Lytic phages lyse PA and through specific depolymerizing enzymes degrade bacterial biofilm thus efficiently killing the host bacteria (Supplementary Table 2) (Criscuolo et al., 2017). What we do not know is which ready-to-use (*prêt-à-porter*) naturally-occurring (ancestral) or trained (evolved) phages or phages isolated on specific patient PA strains (*sur-mesure*, personalized approach) alone or in cocktails could increase phage virulence (lytic activity) against CF PA tested *in vitro* and *in vivo* (Pirnay et al., 2011; Chan et al., 2013; Hraiech et al., 2015). Other open questions include phage gene function, lifecycles, and phage-pathogen interactions. We also need more information on which characterized and genome-sequenced lytic phages, or trained phages or phages stored in therapeutic commercial mixtures, certified as safe from central phage banks or libraries could safely combat MDR PA strains in patients with CF (Sarhan and Azzazy, 2015; Krylov et al., 2016).

Although the Food and Drug Administration recommends lytic phages (Brüssow, 2012), before testing *in vivo* clinical efficacy in CF PA, we lack essential information on the property criteria that should be reported to justify choosing one phage rather than another (Abedon, 2017a). These criteria address methods to assess *in vitro* phage functional characterization (burst size, latency period), activity (host range assay used), bio-informatics genomics (accession numbers from gene sequencing databases, International nucleotide sequence database consortium, INSDC)^1^, how to select and test bacterial hosts to avoid prophage-induced contamination in phage stocks, phage stability, efficacy, and bacterial host resistance *in vitro* and *in vivo* (Azeredo and Sutherland, 2008; Ryan et al., 2011; Jassim and Limoges, 2014; Hraiech et al., 2015; Weber-Dabrowska et al., 2016). Criteria for assessing *in vivo* phage safety include also experiments testing removal (purity) of lipopolysaccharides (LPS) derived from PA lysis, and phage administration routes and doses (Abedon, 2017a).

Although the first randomized controlled trial (RCT) provided evidence on phage therapy in patients with MDR PA-related chronic otitis (Wright et al., 2009), and CF MDR PA is a typical candidate for phage therapy, no previous reviews have addressed this topic. Nor are trials underway on MDR PA in CF. Pivotal studies on genomic technologies theorized that genomic engineered phages, genetically manipulated to modify their host range and induce specific depolymerase biofilm-degrading enzymes, interact better than their naturally-occurring species to lyse antibiotic-resistant bacterial hosts (Lu and Collins, 2007; Edgar et al., 2012; Pei and Lamas-Samanamud, 2014; Reardon, 2014; Salmond and Fineran, 2015; Pires et al., 2016a). Equally important, convincing clinical evidence suggests that combining phages with antibiotics restores antibiotic sensitivity in various MDR PA strains (evolutionary synergy) (Escobar-Páramo et al., 2012; Chan et al., 2016). We, therefore, need experiments to select phages that use as receptors the bacterial membrane zones responsible for expelling antibiotics (multidrug efflux systems) (Chaturongakul and Ounjai, 2014; Chan et al., 2016; Torres-Barceló and Hochberg, 2016; Chaudhry et al., 2017).

Our several-year-long multicenter experience on microbiology in patients with CF (Pollini et al., 2011; Bacci et al., 2016; Moretti et al., 2017), and our promising preliminary observations on phages from a collection stored at Bambino Gesù Children's Hospital tested against mucoid and non-mucoid clinical CF MDR PA strains, prompted us to update our knowledge on phage therapy in CF PA strains. Gaining crucial information on phage pedigree properties (Abedon, 2017a), standardization and integration into models of drug development, and on potential phage therapy in CF (Chan et al., 2013) from reliable and repeatable experiments on phage therapy in combating CF PA, could direct phase III RCTs for testing phage efficacy and safety in hospital settings.

We designed this argumentative review to seek up-to-date reliable information on *in vitro* and *in vivo* evidence for and against lytic phage efficacy in various PA strains useful for planning future research into phage therapy for patients with CF. To do so we reviewed, appraised and synthesized information from publications describing lytic phages tested *in vitro* in PA from various sources, and phage activity, resistance and safety *in vivo* on non-pulmonary and pulmonary host models infected by various laboratory and clinical CF PA strains.

## Methods

**Figure 1:**
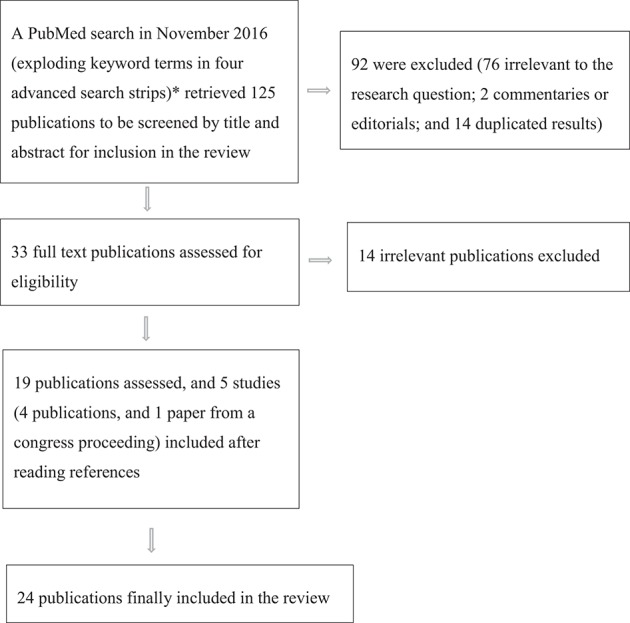
A flow chart showing multiple search strategies and reasons for excluding and including publications in the review. ^*^Four search strategies (November 2016): (((phage) OR bacteriophage) AND cystic fibrosis) AND *Pseudomonas aeruginosa* [TI] (92 publications); ((((phage therapy) AND bacteriophage therapy)) AND *Pseudomonas aeruginosa*) AND cystic fibrosis [TI] (16 publications); ((cystic fibrosis) AND ((phage therapy) AND bacteriophage therapy)) AND *Pseudomonas aeruginosa* biofilm [TI] (6 publications); and ((((phage therapy) AND bacteriophage therapy)) AND *Pseudomonas aeruginosa*) AND phage resistant^*^ (11 publications).

**Figure 2:**
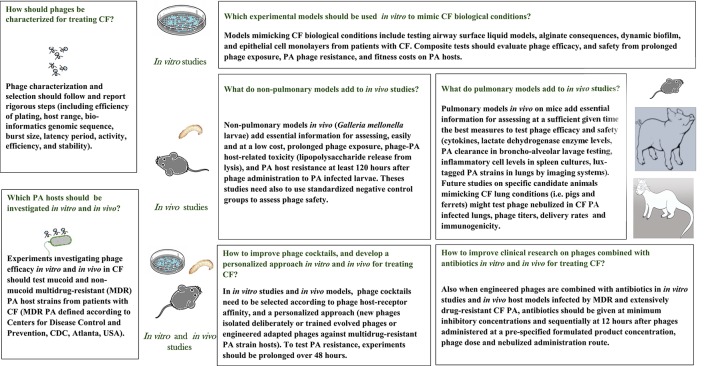
Information from the 24 studies included in the review answering major open questions essential for planning future studies *in vitro* and *in vivo* on phage therapy to treat *Pseudomonas aeruginosa* (PA) lung infections in patients with cystic fibrosis (CF).

In November 2016, to seek papers to include in our review on phage lytic activity *in vitro* and *in vivo* in treating CF PA, we used a multiple sensitive search strategy in PubMed and in a meta-engine (TRIPDATABASE, https://www.tripdatabase.com/), without language, country, and time limits. After retrieving 125 publications, two authors (EVF and PR) screened them by title and abstract, excluded 94 publications, mainly because they were irrelevant to the review question, retrieved and assessed 33 full texts for eligibility, and included 24 publications in the review (Figure 1). Two authors MR and PR mapped and coded information from the papers included using the same author terms (Supplementary Data Sheets 1, 2), and agreed on reporting eight items in *in vitro* studies, and 11 items in *in vivo* studies, according to our experience, and published suggestions on what research should report (Abedon, 2017a). They then identified lines of arguments in key results, problems, and limitations. All three authors discussed key results, weighted advantages and disadvantages in study findings, and combined the synthesis to gain major information essential for planning and advancing future clinical research in CF (Supplementary Tables 3, 4; Figure 2).

## Results

Of the 24 publications included in the review, 14 referred to experiments *in vitro*, 4 to studies *in vivo*, and 5 to studies *in vitro* and *in vivo* (20 studies *in vitro*, and 9 *in vivo*; Supplementary Tables 3, 4). Of the 20 publications describing experiments *in vitro*, 14 were conducted in Western European countries, 3 in Eastern European countries, 2 in the Far East, and 1 in the USA. Although our research fixed no time limit, all the studies included in the review were published in the past 16 years. Only one early study published in 2001 reported the use of a lysogenic phage, unsuitable in human trials (Hanlon et al., 2001). Of the 24 papers reviewed, only 10 specified the criteria used to justify how they selected phages: 6 selected them according to their range of activity (Garbe et al., 2010; Morello et al., 2011; Pires et al., 2011; Alves et al., 2015; Olszak et al., 2015; Lehman et al., 2016), 1 according to previous studies (Friman et al., 2016), 1 using custom-developed phages against resistant PA strains (Essoh et al., 2013), 1 using custom-developed phages against a laboratory PA (Henry et al., 2013), and 1 according to phage ability to delay and inhibit phage-resistant cells (Pabary et al., 2016). Our review, therefore, failed to identify a standardized method for justifying phage selection.

Only three studies *in vitro* reported phage functional characterization estimated by latent phase and burst size (the mean number of phages released per bacterial cell; Garbe et al., 2010; Danis-Wlodarczyk et al., 2015, 2016). Only two studies *in vitro* selected and tested phage activity and host range by efficiency of plating (EOP) (Henry et al., 2013; Uchiyama et al., 2016), 11 used plaque assays, spot test or streak test (Garbe et al., 2010; Pires et al., 2011; Alemayehu et al., 2012; Larché et al., 2012; Betts et al., 2013; Essoh et al., 2013; Alves et al., 2015; Danis-Wlodarczyk et al., 2015; Olszak et al., 2015; Friman et al., 2016; Lehman et al., 2016), and seven left the test used unspecified (Supplementary Table 3; Hanlon et al., 2001; Hall et al., 2012; Coulter et al., 2014; Saussereau et al., 2014; Torres-Barceló et al., 2014; Danis-Wlodarczyk et al., 2016; Lim et al., 2016). Of the nine *in vivo* studies, only three used the EOP (Debarbieux et al., 2010; Morello et al., 2011; Henry et al., 2013), and four used plaque assays or spot test (Alemayehu et al., 2012; Olszak et al., 2015; Lehman et al., 2016; Pabary et al., 2016). Two further studies left the test used unspecified (Supplementary Table 4; Beeton et al., 2015; Danis-Wlodarczyk et al., 2016). Of the 24 papers reviewed, 8 studies *in vitro* left phage bank unreported, and we retrieved and reported genomic sequences from online bank accession numbers (Hanlon et al., 2001; Hall et al., 2012; Betts et al., 2013; Coulter et al., 2014; Saussereau et al., 2014; Torres-Barceló et al., 2014; Friman et al., 2016; Lim et al., 2016). For nine papers *in vitro* (Hanlon et al., 2001; Pires et al., 2011; Hall et al., 2012; Larché et al., 2012; Betts et al., 2013; Essoh et al., 2013; Olszak et al., 2015; Friman et al., 2016; Lehman et al., 2016), and three papers *in vivo* (Olszak et al., 2015; Lehman et al., 2016; Pabary et al., 2016) phage genomic sequence data were unreported or completely or partially irretrievable. Only one investigator reported using a phage taken from a standard reference microorganism central certified collection (Coulter et al., 2014). Only three studies reported phage stability assessment on newly-isolated phages alone (Danis-Wlodarczyk et al., 2015, 2016) and in a phage cocktail (Supplementary Tables 3, 4; Lehman et al., 2016). Of the nine studies *in vivo*, six in PA-infected mouse models instilled phages intranasally, whereas the three in larva models delivered phages into the larval hemolymph. Only three of the nine *in vivo* studies reported the formulated products used for administering the phage suspension to host animal models (Supplementary Table 4; Debarbieux et al., 2010; Morello et al., 2011; Alemayehu et al., 2012). Few papers, therefore, gave essential information on phage functional characterization, selection, activity, genomic sequence, stability, and the formulated products used in phage suspensions useful for further studies on phage therapy in CF PA.

Several investigators reported *in vitro* newly-isolated or trained phage lytic efficacy alone or in cocktails in disrupting the biofilm matrix (including dynamic biofilm models), reducing biomass and planktonic cell numbers in laboratory PA, non-CF and CF mucoid, or non-mucoid PA strains, and MDR PA (Hanlon et al., 2001; Morello et al., 2011; Pires et al., 2011; Alemayehu et al., 2012; Larché et al., 2012; Coulter et al., 2014; Alves et al., 2015; Danis-Wlodarczyk et al., 2015, 2016; Lehman et al., 2016), and penetrating PA alginate at a high concentration (Hanlon et al., 2001). Even though phages penetrated alginate, its overproduction in mucoid CF and non-CF PA strains, under simulated CF lung conditions, reduced phage infection efficiency (Supplementary Table 3; Garbe et al., 2010). These findings underlined the risk that phage efficacy could be influenced by alginate overproduction especially important in patients with CF.

During phage treatment against various PA strains, four studies reported immediate phage resistance to ancestral or newly-isolated or trained single phages (including cross-resistance to new phages) within 1 and 5 days (Pires et al., 2011; Betts et al., 2013; Danis-Wlodarczyk et al., 2015; Friman et al., 2016), and only one study reported the right time to test resistance or cross-resistance to trained phages when standard phage training ends (Betts et al., 2013). In 1 of these studies, trained phages halved the percentage of resistant bacterial cells, leading to lower bacterial growth (fitness cost) in chronic than in intermittent PA isolates (Friman et al., 2016). Another four *in vitro* studies investigated PA resistance to phage cocktails (Hall et al., 2012; Essoh et al., 2013; Saussereau et al., 2014; Alves et al., 2015). Although phage cocktails were tested *in vitro* for no more than 48 h, phage cocktails showed less PA resistance than phages used alone (Hall et al., 2012; Alves et al., 2015). One study, using three phage cocktails inducing similar resistance in several CF PA strains, failed to identify the clustered regularly interspaced short palindromic repeat (CRISPR)-Cas protein system (Essoh et al., 2013). In another study, designed as an *ex vivo* study mimicking a clinical condition, Saussereau et al. (2014) reported that CF PA colonies resistant to a phage cocktail were not resistant to phages per se. Only two studies *in vitro* addressed phage resistance in MDR and extensively-drug-resistant (XDR) PA (some from CF or unspecified origin; Larché et al., 2012; Lehman et al., 2016). Another *in vitro* study reported that combining simultaneously a single phage with a bactericidal antibiotic (tobramycin), PA resistance decreased (Coulter et al., 2014). No study investigated resistance to phages combined with antibiotics for CF MDR or XDR PA infections (Supplementary Table 3; Figure 2). Collectively, few studies reported CF PA phage resistance on phages alone or in cocktails, which have high PA susceptibility. These findings gave no help on selecting, and personalizing phages to use in cocktails to combat chronic CF PA infections.

Of the nine papers describing phage efficiency in *in vivo* studies, three used non-pulmonary host larvae models (*Galleria mellonella*) (Beeton et al., 2015; Olszak et al., 2015; Danis-Wlodarczyk et al., 2016), and six pulmonary models (mice) (Supplementary Table 4; Figure 2; Debarbieux et al., 2010; Morello et al., 2011; Alemayehu et al., 2012; Henry et al., 2013; Lehman et al., 2016; Pabary et al., 2016). Only one of the three studies on non-pulmonary models, tested both single phages and a phage cocktail in non-CF and CF PA-infected larvae, and assessed adverse events from phage injection testing a standardized negative control group, PA uninfected larvae (Olszak et al., 2015). All these studies highlighted the need to prolong PA-infected larvae exposure to phages, given singly or in cocktails, for up to 120 h. Few studies on *in vivo* non-pulmonary models provided reliable findings on phage efficiency, adverse events and host survival, which PA should be selected to avoid massive toxic compound release, or which standardized negative control groups should be assessed to clarify safety from phage injection (Supplementary Table 4).

The six papers testing phages *in vivo* on mouse PA-infected host models mimicked pulmonary conditions (Supplementary Table 4; Debarbieux et al., 2010; Morello et al., 2011; Alemayehu et al., 2012; Henry et al., 2013; Lehman et al., 2016; Pabary et al., 2016). In three studies the investigators proved phage efficacy in preventive and curative mouse models (Debarbieux et al., 2010; Morello et al., 2011; Pabary et al., 2016). In the preventive model, they showed that single ancestral and trained phages prevented PA lung infection. In the curative model, by quantifying imaging (PAKlumi) and bronchoalveolar lavage (BAL) PA counts, they showed that PA loads decreased, rescuing 100% of mice (Debarbieux et al., 2010; Morello et al., 2011). Of the six studies using mouse host models, 4 administering phage-cocktails (two or more phages) to mouse lung infected by various PA strains, all reported that phage cocktails reduced PA loads (Alemayehu et al., 2012; Henry et al., 2013; Lehman et al., 2016; Pabary et al., 2016) and one, comparing PA-infected phage-treated mice with a group of PA-infected mice alone, also showed reduced inflammatory markers in BALs (Pabary et al., 2016). One of these four studies, provided an index to predict phage efficacy reliably especially for phages isolated with a personalized approach (Henry et al., 2013). No study tested *in vivo* phages in cocktails on MDR CF PA, or phage therapy in other animals (pigs and ferrets), possibly better candidates than mice to mimick pulmonary models. Collectively the four studies testing efficiency for 2–5 phage cocktails in various CF and non-CF PA strains gave inconclusive results (Supplementary Table 4).

## Phages combined with antibiotics in *in vitro* studies

Only three studies *in vitro* and no studies *in vivo* investigated whether a single phage combined with various antibiotics (tobramycin, colistin, and streptomycin) reduced a laboratory PA biomass or planktonic cells (Coulter et al., 2014; Torres-Barceló et al., 2014; Danis-Wlodarczyk et al., 2016). Although Coulter et al. and Torres-Barceló et al. showed that combined therapy was as effective as the tested antibiotics alone, the combined treatment reduced the emergence of phage PA-resistant cells. In their study, Torres-Barceló et al. also recommended ensuring maximum antibacterial efficacy, by giving antibiotics 12 h after phages rather than simultaneously and obtained a synergistic effect in decreasing laboratory PA biofilm biomass or density. Testing a single phage on several strains, Danis-Wlodarczyk et al. showed colistin antagonism to the phage (Supplementary Table 3; Figure 2). No study tested phage cocktails combined with antibiotics. In defining experimental efficiency for phages and antibiotics combined, no study mentioned essential information such as host receptor affinity, personalized approaches, well-selected phage cocktails, antibiotics selected by minimal inhibitory concentration (MIC) *in vitro* on MDR PA, or *in vivo* on PA-infected animal host models.

## Problems related to phage efficacy and safety in *in vitro* and *in vivo* sudies

Of the 24 studies, only 14 reported experimental problems (10 *in vitro* and 4 *in vivo*). Three investigators reported *in vitro* problems related to PA biofilm: bacterial regrowth (Pires et al., 2011), increased PA biomass in the phage-treated group (Alves et al., 2015), and PAO1 phage insensitivity (Supplementary Table 3; Danis-Wlodarczyk et al., 2015). Although Pires et al. (2011) suggested overcoming these problems by using short-term phage therapy, preferably in cocktails to avoid PA resistance, no other studies mentioned possible solutions. Four groups reported *in vivo* phage inefficacy including genetically-related phage inability to lyse the same PA strain (Henry et al., 2013), *G. mellonella* larval death several hours after phage infection (Beeton et al., 2015; Olszak et al., 2015), and phage inefficacy in the delayed and prophylactic approaches (Supplementary Table 4; Pabary et al., 2016). In their *in vivo* study in a larva model, Olszak et al. to infect larvae, before phage infection, gave weakly virulent PA strains at high loads, and consequent PA lysis caused massive toxic compound release followed by larvae death at 96 h. Although no study suggested how to overcome problems in *in vivo* phage therapy, Olszak et al. (2015) recommended carefully selecting phages. Finally, although Torres-Barceló et al. (2014) showed a synergistic effect using a single phage combined with an antibiotic (streptomycin), Danis-Wlodarczyk et al. (2016) detected colistin and phage antagonism. Few investigators suggested how to overcome problems related to efficacy and safety in phage treatment or by using a phage combined with antibiotics in CF PA. None of the studies we reviewed compared phage efficacy *in vitro* or *in vivo* according to administration routes.

## Discussion

Our argumentative review provides critically appraised, up-to-date previously unavailable information for, and against, *in vitro* and *in vivo* lytic phage efficacy in various CF PA strains, useful for planning future clinical research into phage therapy for patients with CF (Supplementary Tables 3, 4; Figure 2). Although our multiple search strategy avoided language and time limits, the most promising and pertinent information came mainly from studies published by eminent research groups around the world over the past seven years, presumably owing to MDR PA spread, and disinterest in producing new antibacterial drugs (Salmond and Fineran, 2015; Krylov et al., 2016). By collecting information from 24 relevant publications describing lytic phage activity and efficacy *in vitro*, and efficacy and safety *in vivo* experiments on pulmonary and non-pulmonary host models infected with PA from various sources, our review fills some major knowledge gaps on phages that can effectively combat PA strains infecting lungs in patients with CF. Our findings also provide new insights that will prompt renewed clinical research in CF PA to test well-selected (ancestral or trained) lytic phages to include in cocktails, develop personalized phage therapy, address phage-antibiotic combinations, and envisage even genomic engineered phages combined with antibiotics to treat MDR and XDR PA in patients with CF (Figure 2).

Among research that especially takes the field ahead in phage treatment for CF PA, our review provides useful information on the various methods used for testing host range and lytic phage activity against a variety of target bacteria (spot tests, plaque assays, and EOP). Ample evidence suggests that the essential, reliable step for confirming lytic phage cocktail efficiency as preliminarily tested by spot test or plaque assay, is to test single phages for effectiveness with EOP (Supplementary Tables 3, 4; Figure 2; Debarbieux et al., 2010; Morello et al., 2011; Henry et al., 2013; Uchiyama et al., 2016). Leaving EOP *in vitro* unassessed, as many papers in our review did, risks selecting phages that despite their broad lytic activity, as assessed *in vitro* by other methods (spot tests that simply reflect a bactericidal effect, or plaque assays), fail to infect CF PA planktonic cells (Pires et al., 2011). Surprisingly, among the nine *in vivo* studies reviewed, three studies conducted in the past two years left methods for assessing lytic phage host range unspecified or unreported (Beeton et al., 2015; Danis-Wlodarczyk et al., 2016; Lehman et al., 2016), and three reported only plaque assay or spot tests (Alemayehu et al., 2012; Olszak et al., 2015; Pabary et al., 2016). As Henry et al. (2013) underlined, EOP for seven over nine phages correlates *in vitro* with activity *in vivo* in PA-infected mice, thus predicting lytic phage efficacy. These findings were later confirmed by two investigators who concluded that EOP is the standard method also for confirming lytic phage activity against other Gram-negative bacteria, namely *Escherichia coli* and *Salmonella* reference collections (Mirzaei and Nilsson, 2015).

A key problem in ensuring the efficacy of newly-isolated lytic phages in cocktails is selecting and mixing specific phages having high lytic activity that can also disrupt and reduce the CF PA biofilm matrix, by producing or inducing biofilm matrix-degrading enzymes (Harper et al., 2014; Pires et al., 2016b). In biofilm aggregates, enzymes produced by the CF PA community breakdown the polysaccharides that hold the biofilm together, thus actively releasing surface bacteria (planktonic cells) that colonize fresh substrates (Supplementary Table 1). In our review, two studies reported that two different 2-phage cocktails disrupt and reduce, though by only 2-log (Alemayehu et al., 2012) the CF PA biofilm, also showing age-independent biofilm efficacy (Danis-Wlodarczyk et al., 2015). Phage cocktails, probably by mixing specific biofilm-exopolysaccharide-degrading enzymes, increase lytic phage efficacy by reducing bacterial population densities (Hall et al., 2012), and preventing bacterial resistance (Supplementary Table 3; Alves et al., 2015). Overall these findings imply that phage cocktail therapy might be useful in preventing PA lung colonization, and in reducing pulmonary exacerbations in patients with CF (Anderson, 2012). Accordingly, new evidence confirming that phage cocktail efficacy depends on selecting the correct phage-degrading-enzyme mixtures comes also from those who claim that in clinical practice phage therapy will probably have limited usefulness in eliminating bacterial infections caused by biofilm aggregates, i.e., microbial communities inhabiting chronic infections. Conversely, phage therapy could be of great use in reducing cells that detach themselves from the biofilm and revert to the virulent planktonic phenotype (Criscuolo et al., 2017; Darch et al., 2017). Another additional benefit from phage therapy comes from a study included in our review showing reduced inflammatory markers in BALs from PA-infected phage-treated mice compared with controls (Pabary et al., 2016), as others confirm in a mouse model infected by pulmonary *Burkolderia cenocepacia* (Carmody et al., 2010).

Another major *in vitro* and *in vivo* experimental requirement related to formulating efficient newly-isolated phage cocktails to combat PA lung infection in patients with CF is to select highly virulent lytic phages having good, varying target host-receptor affinity (Figure 2). Choosing phages that differ in host receptor affinity avoids competition in absorption to the bacterial surface, and improves synergic phage cocktail efficacy (Drulis-Kawa et al., 2012). Hence, surprisingly, among the 24 papers on CF PA reviewed, only one underlines the need to select phages according to their host receptor affinity, to avoid antagonism, and ensure a phage synergistic effect (Supplementary Table 3; Essoh et al., 2013). This information is particularly lacking in the otherwise highly informative *in vivo* study conducted by Olszak et al. (2015) in which the phage mixture probably competed for bacterial receptors (Supplementary Table 4).

Our review also provides new insights into CF PA resistance, including defense mechanisms that allow phages to adapt to changing host systems, thus incurring significant fitness costs. One study in our review (although it failed to test resistance on a CF PA strain) emphasizes that a phage-cocktail resistant PAO1 emerges in experiments investigating simultaneous and sequential phage-cocktail exposure. In this study, fitness costs probably arose when phage-cocktail resistant PA overproduced alginate or extracellular polymeric substances (EPS) (Hall et al., 2012). How PA phage resistance develops in CF or how PA naturally alters its genotype or phenotype to combat phage virulence is debatable (Mendes et al., 2014; Krylov et al., 2016). Bacteria have developed an astonishing array of defense strategies to combat phages at each step during the infection. These include adsorption-blocking systems, systems for preventing phage DNA entry into the host cell, bacterial restriction-modification systems, phage abortive infection, and genetically modified systems such as the renowned CRISPR-Cas protein system (Labrie et al., 2010). The bacterial CRISPR-Cas system aims to inactivate invading phage genetic material. Although a study conducted in recent years suggests that many clinical PA isolates develop the CRISPR-Cas system (Cady et al., 2012), in our review Essoh et al. (2013) observed in the 13 phage-resistant PA strains studied from different clusters isolated from patients with CF no CRISPR-Cas system (Supplementary Table 3). Although Bondy-Denomy et al. (2013) underline that specific phage genes inactivate the CRISPR-Cas system, our review on CF PA lacks information on phage-borne CRISPR inactivation systems.

Besides using phages in cocktails to treat CF PA, another strategy for overcoming PA resistance in patients with CF includes phage training. Only one *in vivo* study (Morello et al., 2011) reported that trained (man-guided evolved) phages were more efficient than untrained (ancestral) phages in killing CF MDR mucoid PA lung-infected mice, and in reducing bacterial resistance *in vivo*. They also observed that in the curative phage treatment model, trained phages and ancestral phages act cooperatively with the mouse immune response to eliminate MDR PA acute lung infections. Others subsequently highlighted contrasting results, and possible drawbacks in using trained phages *in vitro* (Supplementary Table 3, 4; Betts et al., 2013; Friman et al., 2016). Whereas Betts et al. observed that during training bacteria exposed to phage can, in turn, evolve cross-resistance toward ancestral and newly-isolated phages, Friman et al. showed that evolved phages were more efficient than untrained phages in killing CF PA strains, and in reducing bacterial resistance. Underlining an equally important problem, Henry et al. (2013) demonstrated that phage training is not always feasible, stating in their *in vivo* study that they were unable to evolve a highly-virulent phage. Insofar as phage optimization by only two single nucleotide mutations extends host range and is sufficient to improve phage virulence toward CF MDR PA clinical strains (Morello et al., 2011), trained phages possess broad lytic activity and virulence, and might be useful in advancing future research on phage genetic changes (Figure 2).

Whether lytic phages can be used to prevent as well as treat PA infections in patients with CF remains unclear until we have long-term results, and use pulmonary models on the best animal candidates. Of the 24 studies reviewed, two showed that preventive treatment requires not heat-killed but active phages (Debarbieux et al., 2010; Morello et al., 2011), thus providing evidence that phage efficiency depends on phage lifecycles. Of the nine *in vivo* studies we reviewed, four investigated phage treatments for preventing PA infections (Debarbieux et al., 2010; Morello et al., 2011; Beeton et al., 2015; Pabary et al., 2016). Despite the impressive efficacy reported, some investigators underline that the preventive approach is of minor clinical interest because it cannot predict the specific PA strain eventually infecting the patient (Morello et al., 2011). Another open question is whether phage therapy can eradicate MDR PA from the airways in patients with CF. Findings in recent years provide evidence that phage therapy can prevent the high-density MDR PA increase and antibiotic treatment failure (Chan et al., 2016; Torres-Barceló and Hochberg, 2016; Chaudhry et al., 2017). A significant and sustained reduction in the number and activity of infecting PA could undoubtedly improve clinical conditions in patients with CF (Krylov, 2014).

More information advancing clinical microbiological research in CF, and optimizing phage-cocktail efficacy in treating PA concerns the need to personalize phage therapy by using a standardized approach. Two papers in our review underline that the most efficacious phages are those isolated on purpose (personalized phage cocktails) against the patients' infecting, and eventually antibiotic-resistant PA strains (*sur-mesure*, patient-tailored therapy; Henry et al., 2013; Saussereau et al., 2014). Yet despite its advantages, the personalized approach has several drawbacks because it is time consuming (phages need to be isolated, purified and completely characterized). Difficulties also arise in assessing phage effectiveness on PA strains isolated in certain medical facilities, and from ḡenetically poorly-differentiated local clones, thus introducing subjectivity in assessing phage lytic activity (Krylov et al., 2016). The lytic phages described in our review, and in current practice, often lack detailed criteria for justifying phages chosen for experiments, their genomic sequences, and database accession numbers. These problems reduce the chances for selecting an optimal set of lytic phages to mix in a cocktail, impede experimental repeatability, and make it hard to generalize the results (Supplementary Tables 3, 4; Figure 2; Abedon, 2017b; Adriaenssens and Brister, 2017).

Our review leaves unclear whether and to what extent phage efficacy depends on administration routes. No studies have compared the various phage administration routes in treating MDR PA or CF PA *in vitro* or *in vivo* models. Although the best way to reach PA-infected host lungs may be by delivering phages as a liquid or a dry powder (Carrigy et al., 2017; Chang et al., 2017), the murine CF PA-lung-infected models we reviewed only used intranasal instillation (Debarbieux et al., 2010; Morello et al., 2011; Alemayehu et al., 2012; Henry et al., 2013; Lehman et al., 2016; Pabary et al., 2016). New studies need to address the potential benefits from giving phage by inhalation instead of intranasally. What we mainly need to know is which device to choose for phage inhalation and the right animal models for reproducing human CF airways anatomically and physiologically (Lavelle et al., 2016). Only one study *in vitro* has compared two nebulizers and one inhaler to define the best active liquid phage delivery in treating *Mycobacterium tuberculosis*. The investigators concluded that delivering phages with a vibrating mesh nebulizer is significantly more effective than using a jet nebulizer (*p* < 0.01), whereas a soft mist inhaler may be useful for self-administration of a phage aerosol (Carrigy et al., 2017). Apart from the type of device, another important question to address is the time for nebulizing phages. Known mathematical models can help to assess the best time for nebulizing so as to increase PA clearance from the airways (Nadithe et al., 2003). Although again in a murine model, new information in a non-comparative study on an MDR PA-neutropenic lung-infected mouse model suggests that a dry powder phage formulation might have advantages over a liquid formulation, and could be applied also in CF MDR PA-infected animals (Chang et al., 2017). New promising information advancing *in vivo* experiments overcoming murine model limitations (no mucus hypersecretion in mice, and the cystic fibrosis transport regulator, CFTR, gene is expressed only in the proximal trachea) envisages specific animal candidates (pigs and ferrets; Lavelle et al., 2016). Like humans, these animals exhibit the same high CFTR expression in serous cells of submucosal glands in the cartilagineous airways, thus mimicking CF lungs. By using these experimental procedures in *in vivo* models, to deliver the best sufficient active phage titers for targeting MDR CF PA in the lungs, we could also address phage pharmacokinetics (Trend et al., 2017). Similarly, by using various phage administration routes to address efficacy on combating MDR CF PA lung infections, and negative or adverse phage effects, we could also investigate another unaddressed problem, phage pharmacodynamics (Abedon, 2017a). Pharmacokinetics and pharmacodynamics can help transfer research results into clinical care. Even more important, we need to test *in vivo* phage adverse effects related to immunogenicity in pig and ferret models in studies with a follow-up long enough to allow chronic pulmonary inflammation determined by bacterial persistence to develop (Figure 2; Lavelle et al., 2016; Lin et al., 2017; Trend et al., 2017). Pig and ferret models can also address research on nebulized phage treatment to test what might happen in immune interference from innate immune responses, neutrophil elastase, secretory IgA and macrophages engulfing phage particles after repeated phage administration in CF lungs (Trend et al., 2017).

Another problem that has in the past discouraged research on phage therapy in general clinical use, is how to define phage doses to test phage safety. Our review, thanks to the published studies *in vivo*, gives up-to-date information for designing future CF PA clinical studies possibly avoiding risky phage-induced reactions and phage-PA interactions. Many phages collected in libraries and several phages in the studies included in our review came from human waste (sewage; Supplementary Data Sheets 1, 2). As human beings, we normally encounter phages throughout our lifetime, owing to the complex interactions between bacteria and phage in our colon, upper respiratory system, and on our skin (Merril et al., 2003). In assessing safety *in vivo*, to investigate possible adverse events we therefore need to include standard negative control groups (PA-uninfected or phage-uninfected animals). For example, experiments testing sham solution injected or nebulized in PA-and-phage-uninfected animals can detect injection-related adverse events, whereas those testing PA-uninfected and phage-treated animals investigate potential phage virulence. Surprisingly, of the nine *in vivo* studies examined in this review six used unstandardized negative control groups, and reported no safety problems, and only one study assessed safety with standardized negative control groups (Supplementary Table 4; Olszak et al., 2015). This major study also investigated safety *in vivo* in positive (PA-infected) larvae at high CF PA loads. An unexpected result was that the weakly-virulent PA infected larvae required a higher lethal dose than the highly-virulent PA (10^5^ vs. 10), and more phage inoculum to keep the multiplicity of infection (MOI) value of 100. They reported the highest mortality at 48 h in the weakly virulent CF PA owing to toxic compounds released by bacterial lysis. In their study Olszak et al. (2015) also used inactivated phages to treat larvae infected with PA strains at a lethal dose, and showed no differences in larvae mortality compared with control infected caterpillars, thus confirming that larval survival was entirely due to phage lytic activity rather than to host immune stimulation (Supplementary Table 4). In a previous study, Debarbieux et al. (2010) gave bioluminescent-non-mucoid non-CF PAK-infected mice a high phage concentration, monitored their behavior for 10 days, and again reported no adverse effects (Supplementary Table 4). Similar results came from a study conducted by Wang and colleagues in bacteremic mice infected with an imipenem-resistant PA strain, who concluded that inoculating phages at high doses produced no adverse effects in mice (Wang et al., 2006). In the same experiment, Wang et al. underline that to avoid severe adverse effects potentially caused by microbial debris, including the endotoxin LPS released during phage propagation, phages prepared for therapeutic applications must be highly purified (Biswas et al., 2002; Chan et al., 2013). A possible solution to avoid LPS-related mortality, rather than using phages having a high burst size (the average number of phages produced by infected cells) thus producing many virions guaranteeing that sufficient phage numbers reach the infection site (Mirzaei and Nilsson, 2015), in patients with CF we suggest selecting a poorly virulent phage cocktail, with a low burst size nebulized directly to the infection site. Giving patients nebulized drugs to treat lung infections is a standardized procedure in routine clinical practice. Ample evidence shows that phages can be nebulized or delivered as a dry powder (Golshahi et al., 2011; Matinkhoo et al., 2011; Sahota et al., 2015). We now await information from tests using a powdered preparation including two phages (phiKZ, active on PA, and KS4-M, lysing *Burkholderia cepacia*) given by inhalation to patients with mixed infections (Golshahi et al., 2011). If inhaled phages reach the infection site directly, possible characterized and selected phage candidates might include those having a low burst size, thus causing the infection to progress at a slower rate, hence probably diluting LPS release over time.

Another concern, insofar as efficacious phage therapy involves giving active, vital virus (Morello et al., 2011; Olszak et al., 2015), is the need to assess whether treatment remains effective and safe over time. For example, it should avoid complications related to phage-resistant hosts, bacteria whose pathogenicity has been altered by transduction phenomena, and phage-host genetic rearrangements (Krylov, 2014). Although these deleterious events are extremely rare, they might develop especially during the prolonged phage therapy needed for a chronic disease (Waddell et al., 2009). Hence the need to use phage mixtures delivered from certified reserve storage (phage banks or libraries) including characterized phages having a wide host range, and differing in PA receptor affinity (Domingo-Calap et al., 2016). These phage mixtures could become an extremely valuable clinical resource for ensuring safety, should help laboratory clinicians rapidly to select appropriate phages to include in cocktails, and possibly adapt cocktails to the MDR PA strains isolated during CF pulmonary exacerbations (Figure 2; Krylov et al., 2016). Even though our review included only one study that specified the use of a phage from a certified bank (Coulter et al., 2014), and although phage banks lack international institutional regulations, our review identified no information or data on a promising new research direction for clinical phage therapy envisaging combining phage cocktails from individual researchers' phage banks or phage libraries, and a personalized approach using phages isolated on CF PA strains. An alternative solution to phage libraries for testing and selecting personalized phage-cocktails, taking into account the extremely high PA genome plasticity (Supplementary Table 1), might be to set up an international reference library based on clinical CF PA strains with various characteristics (Shen et al., 2006; Riou et al., 2010; Krylov, 2014). Such presumptive phage selection on an internationally available PA reference set also lacks international regulations including well-combined and continuously updated newly-isolated lytic phages. An intriguing modular approach proposed in 2007 (Lu and Collins, 2007) and reported also by Krylov et al. in 2016 entails formulating phage mixtures (mono-species or hetero-species), enhancing their activity by introducing newly characterized engineered phages to express the most effective EPS-degrading enzymes specific to the target biofilm, and combining them in a personalized way to treat PA infection in patients with CF. This modular approach and the mixtures included in phage libraries could help international regulatory agencies to address phage therapy in CF. For example, it could allow different research groups to cooperate in developing a central phage library, to ensure distributing phage mix on call, thus encouraging the use of the phage therapy in untreatable CF XDR PA pulmonary infections (Figure 2; Krylov et al., 2016). Prompted by these promising results, to receive more information on their studies on phages used in clinical therapy in Eastern countries, including sequence information useful for determining whether some phages used in various studies belong to the same or related clusters, while collecting data for our review we contacted by email Krylov, and after receiving his advice, contacted two other authors (Shabalowa and Karpanov). Both answered in brief, specifying that they will report their findings in published papers, but failed to give more information. Hence, we were unable to compare phage efficacy across different studies. Although disseminating detailed unpublished research data could be a challenging matter, phage libraries are expensive, need to be continuously updated to account for variability in target bacteria, and can vary from region to region (Chan et al., 2013).

Considering the known resistance to a three-phage cocktail in MDR PA and XDR PA (Larché et al., 2012), another promising approach that helps in reducing MDR PA biofilms, as well as the PA planktonic cells responsible for exacerbations in CF, is to improve treatment effectiveness by combining phage therapy and antibiotics (Figure 2). Although combined phage-antibiotic treatment started when the antibiotic era began (Himmelweit, 1945), questions remain on whether to use sequential treatment, optimal phage administration routes, dosing, suspended formulated products, and treatment duration. Surprisingly, only three studies in our review investigated phage therapy combined with tobramycin, streptomycin and colistin on laboratory PAO1 (Coulter et al., 2014; Torres-Barceló et al., 2014; Danis-Wlodarczyk et al., 2016). The best strategy to ensure phage efficacy, allow a wide antibiotic choice, and minimize possible antibiotic antagonism and PA resistance, is to apply antibiotics at peak phage efficacy (within about 12 h) sequentially rather than simultaneously (Supplementary Table 3; Figure 2; Torres-Barceló et al., 2014), as recently confirmed (Chaudhry et al., 2017). We retrieved no studies testing other antibiotics (aminoglycosides, beta-lactams, quinolones, carbapenems or macrolides) combined with phage against CF and non-CF PA strains, and none investigated phage therapy combined with antibiotics for CF XDR PA strain infections *in vitro* or *in vivo*. We remain skeptical about why Danis-Wlodarczyk et al. (2016) combined phage therapy with colistin, a drug known to alter the bacterial membrane thereby removing phage bacterial receptors (Martis et al., 2014). No comparative head-to-head studies reported antibiotic therapy and trained phages as alternative treatments in CF PA strains. Overall, the promising findings this review highlights on trained (man-guided) phages and on how certain antibiotics effectively combat CF MDR PA and new preliminary information suggesting that the amikacin-phage combination could have potentially more benefits on PA biofilms than meropenem (Nouraldin et al., 2016) lead us to conclude that trained or even engineered phages combined with amikacin or meropenem at sub-inhibitory concentrations, might reduce PA resistance, thus boosting interest in phage therapy (Figure 2). The evolutionary rationale for combining phages with antibiotics provides support for designing clinical trials using combined phage-antibiotic therapy possibly in skin infections, such as diabetic ulcers, before applying the best results to systemic diseases in humans (Torres-Barceló and Hochberg, 2016; Chaudhry et al., 2017). New evidence implies that genetically engineered phages that might act against PA biofilms better than newly-isolated phages, expand their host range and, by so far undefined co-evolutionary phage-bacteria interactions, possibly sensitize MDR and XDR PA antibiotic efficacy (Pires et al., 2015; Salmond and Fineran, 2015; Chan et al., 2016; Torres-Barceló and Hochberg, 2016). Again underlining the need for combining phage with antibiotics in treating CF MDR PA, a future crucial research step, before undertaking *in vivo* therapy, must include pre-testing antibiotics to assess the standardized MIC for MDR PA isolates. Other essential stages include characterizing and selecting phages assessed for host range by EOP, by host receptor affinity, and by a personalized approach. Final steps include choosing phages also from phage libraries for formulating well-designed phage cocktails, and conducting research on antibiotic and phage dosing, timing, and administration routes (Figure 2).

## Future promises on phage therapy against CF PA

Although clinical trials for phage therapy in patients with CF seem a long way off in Western countries (Parracho et al., 2012; Reardon, 2014), the alarming evidence on growing resistance to antibiotics can only encourage efforts to exploit the promising phage therapy for MDR and XDR PA in patients with CF. Researchers responsible for designing future clinical trials for phage therapy in humans will need to choose the correct experimental *in vitro* and *in vivo* models on biofilm-producing mucoid PA (Figure 2; Costerton et al., 2003). As a first step, they should preferably test *in vitro* a mono-microbial-biofilm-forming mucoid PA model or a non-biofilm-forming PA incorporated in biofilm-producing isolates mimicking a CF lung condition (Deligianni et al., 2010). These biofilm and non-biofilm forming PA often coexist in poly-microbial communities in CF lungs (Supplementary Table 1). Equally important, although phages focus their action specifically on the target pathogen (Loc-Carrillo and Abedon, 2011), and although Rogers et al. (2005) state that the various bacteria persist in an active form without interfering with the lung disease or influencing patients' prognosis, others observed that various microbial species present in the biofilm can alter phage-induced responses, influencing bacterial and phage densities, and determining how bacterial resistance evolves (Mumford and Friman, 2017). After identifying the target and the strain to treat, the next step will be to select the most appropriate phages to combine in a cocktail, according to their EOP, their affinity for the various PA receptors, their ability to degrade the biofilm matrix, and their low burst size. Future research on phage administration routes will consider nebulizing phages, suspended in pre-specified material or giving them as a dry powder (Figure 2; Golshahi et al., 2011; Matinkhoo et al., 2011; Sahota et al., 2015).

Another crucial step is to characterize selected phages functionally (latency and burst size) and to detect those carrying undesirable genes coding for toxins and antibiotic resistance (Loc-Carrillo and Abedon, 2011). Some evidence suggests that safety can be efficiently and reliably assessed by separating lytic phages from lysogenic (temperate) phages characterizing them by genome sequencing and analysis (Supplementary Table 2; Chan et al., 2013; Mattila et al., 2015), and by using standardized control groups in *in vivo* studies (Supplementary Table 4; Figure 2). Future directions should include research to investigate effective trained phages in cocktails (Domingo-Calap et al., 2016) or engineered phages for formulating new phage cocktails, even combined with antibiotics (Lu and Collins, 2007). Phage cocktail efficacy and safety, and phages eventually combined with antibiotics, should then be validated *in vivo* in skin diseases before testing them in systemic diseases, and easily at a low cost in non-pulmonary models. Because human immunological responses to phages differ significantly during chronic infections (Li et al., 2005; Lin et al., 2017; Waters et al., 2017), we also need to test outcomes in studies investigating long-term PA infections and biofilm forming PA. Hence our review stresses the challenging need to standardize current biofilm models. What we especially need are dynamic models closely resembling physiological conditions (Alves et al., 2015), or models mimicking PA growth in CF lungs including the mucus barrier, such as the airway surface liquid model used on epithelial cell lines (Danis-Wlodarczyk et al., 2016), artificial sputum medium (Garbe et al., 2010) or eventually patients' sputum (Saussereau et al., 2014), and test *in vivo* specific animal candidate (pig and ferret) models. All experimental procedures should include standardized negative control groups (Figure 2; Olszak et al., 2015).

Future promises and perspectives in phage therapy for treating MDR and XDR PA lung infections in CF include models designed to apply antibiotics to phages at peak phage efficacy (within about 12 h) sequentially rather than simultaneously (Torres-Barceló et al., 2014). We envisage future head-to-head studies comparing antibiotics given at sub-inhibitory concentrations combined with trained phages and antibiotics given alone (Nouraldin et al., 2016; Lin et al., 2017). These research advances might reduce PA resistance, and boost interest in phage therapy in patients with CF (Figure 2).

## Conclusions

In conclusion, our review results addressing practical *in vivo* models for advancing clinical research on phage therapy should help in designing protocols for pivotal clinical trials, to test phage therapy against CF PA strains in close coordination between microbiologists and clinicians caring for patients with CF (Figure 2). It also involves implementing dialogues between the medical community in consultation with CF community reference groups able to overcome complex ethical dilemmas (Caplan, 2015; Trend et al., 2017).

The results from our review suggest using phages with a broad host range against specific infecting PA strains in CF lungs (Alemayehu et al., 2012; Pabary et al., 2016), and avoiding polyvalent phage mixtures (Saussereau et al., 2014; Beeton et al., 2015; Olszak et al., 2015; Lehman et al., 2016). This approach promises clinical advantages including reducing costs, speeding phage cocktail delivery, analyzing dose-response curves, and “weeding out” phages that could show incompatibility (Chan and Abedon, 2012). Although no research has yet defined the kinetic mechanisms responsible for phage actions especially against CF PA biofilms, to simplify phage therapy development we agree with others (Chan and Abedon, 2012; Domingo-Calap et al., 2016) on the need to design clinical protocols investigating a minimum number of active phages (2–3 phages) via a nebulizer or by an inhaler delivered directly to the CF lungs (Carrigy et al., 2017; Trend et al., 2017). This standard way is effective and meets with patients' approval.

Given the importance of simulating *in vivo* a condition resembling that in CF patients' lungs, overall the findings from our review suggest that research needs also to develop models *in vivo* able to detect a change from acute to chronic PA infections in CF, and to investigate the most important concerns on MDR and XDR PA phage co-evolutionary interactions and immunogenicity. Future studies on immunogenicity in animals that exhibit a high CFTR expression in lungs similar to that in CF (pigs and ferrets) will show whether mucus can stimulate phage adhesion to the lungs, prolong phage therapeutic activity, and how phage particles can overcome immune interference (Barr et al., 2013; Trend et al., 2017). To prevent therapeutic phages from spreading outside special clinical facilities designed for applying phage therapy, we agree also with those who recommend using phages propagated only on standard host strains (such as PAO1), and choosing therapeutic phages individually for each patient (personalized approach) (Krylov et al., 2016). A crucial step is to use phage genome sequencing to identify possible virulence factors or prophages from host strains, so as to avoid transferring possible contamination in phage stocks (Figure 2; Abedon, 2017a). Convincing research from synthetic biology and nanotechnology techniques already exploits new engineered phages that display selected proteins binding specific CF PA targets isolated and re-amplified from commercially available phage libraries (Smith, 1985; Kaur et al., 2012; Henry et al., 2015; Criscuolo et al., 2017). Using phages from certified libraries will help to overcome concerns requiring international regulatory agencies to approve and re-approve phage cocktails for each individual clinical trial (Parracho et al., 2012; Salmond and Fineran, 2015; Sarhan and Azzazy, 2015; Criscuolo et al., 2017). Even though obtaining a phage set that effectively lyses all variants in a given pathogen is a challenging problem (Pirnay et al., 2012; Chan et al., 2013), our review findings suggest that successful phage therapy on a larger scale in patients with CF depends not only on involving the pharmaceutical industry in preclinical development and formulation before undertaking clinical trials. Preclinical industry-funded phage formulation could be developed also by creating the claimed first-in-human advisory system (Kimmelman and Federico, 2017). Despite the high investment needed for creating an advisory board regulatory infrastructure, this regulatory board could critically match preclinical evidence and clinical scenarios for phage therapy in CF PA, so as to justify the first-in-human trials for phage therapy, thus meeting a great medical need in treating CF MDR and XDR PA. This new approach will help to improve laboratory techniques intended to develop phages combined with antibiotics able to combat MDR and XDR PA strains (Chan et al., 2013; Domingo-Calap et al., 2016; Torres-Barceló and Hochberg, 2016; Lin et al., 2017). Combining phage therapy with antibiotics might be more appealing to pharmaceutical companies because it gives them opportunities for new patents.

Collectively, the findings from our review argue that, despite the continuing need to improve phage efficacy and safety *in vivo* and solve ethical and regulatory concerns, research into lytic phage therapy in CF PA strains holds promise. Progress over the past 20 years intended especially to combat increasing antibiotic resistance, including hospital studies, already suggests that lytic phage therapy of CF PA infections will become a useful, safe medical procedure. Though phage therapy will never completely replace antibiotics in patients with CF, pharmaceutical industries should eventually combine phages with one or two antibiotics scaled by MIC to pre-test their sensitivity to treat CF MDR and XDR PA. Advances that should encourage and simplify phage therapeutic use in CF MDR and XDR PA include regularly updating cocktail preparations in certified phage libraries for personalized phage therapy, preferably using genomic engineered phages, so as to ensure safety and possibly patentable engineered phages, thus providing certified phages for international regulatory agencies (Figure 2). Researchers should release all available phage therapy results to the medical society so that procedures can be corrected and adjusted as necessary. Owing to easy phage isolation, sequence-specific targeting and replication on specific bacterial hosts, active penetration into bacterial biofilms, low production costs, safety, lack of known immunogenicity, and disinterest in producing new antibiotics, introducing phage therapy combined with existing PA-sensitive antibiotics in treating CF PA in clinical practice appears a promising development over time.

## Author contributions

EF and PR: searched and screened articles by title and abstract; PR and MR: mapped and coded information. All the three authors identified key results, limitations, advantages and disadvantages in study findings, combined the synthesis, wrote and revised the re-submitted paper, and approved the final version.

### Conflict of interest statement

The authors declare that the research was conducted in the absence of any commercial or financial relationships that could be construed as a potential conflict of interest.

## Footnotes

^1^International Nucleotide Sequence Database Consortium [INSDC]. Available online at: http://www.insdc.org/

## Abbreviations

BAL: bronchoalveolar lavage
CF: cystic fibrosis
CFTR: cystic fibrosis transport regulator
CFU: colony-forming units
CRISPR: clustered regularly interspaced short palindromic repeats
EOP: efficiency of plating
MDR: multi-drug-resistant
MIC: minimal inhibitory concentration
MOI: multiplicity of infection
PA: *Pseudomonas aeruginosa*
PBS: phosphate-buffered saline
PFU: plaque-forming units
XDR: extensively-drug-resistant.
