# Supplementary material for: Challenges and Promises for Planning Future Clinical Research Into Bacteriophage Therapy Against Pseudomonas aeruginosa in Cystic Fibrosis. An Argumentative Review
Source: Front Microbiol. 2018 May 4;9:775. doi: 10.3389/fmicb.2018.00775 (PMC5945972; doi:10.3389/fmicb.2018.00775)
Supplement: Supplementary file 1 [file Data_Sheet_1.docx]

| **Additional file 1 \| Line of arguments from 20 studies included in the review investigating *in vitro* lytic bacteriophage (phage) effects against laboratory *Pseudomonas aeruginosa* (PA) strains or non- cystic fibrosis (CF) strains or PA strain hosts isolated from patients’ wounds, patients with diseases other than CF, and patients with CF (studies reported alphabetically according to the first authors’ surnames).** | | | | | | | | |
| --- | --- | --- | --- | --- | --- | --- | --- | --- |
| **First author, year (Country)** | **Phage taxonomy (family)*** | **Phage sources** | **PA strain hosts** | **Procedures** | **Results** | **Problems reported on safety and efficacy** | **Limitations** | **Strengths** |
| 1-Alemayehu, 2012 (Ireland)** | Two newly-isolated φNH-4 (*Myoviridae*) and φMR299-2 (*Podoviridae*) | Sample from sewage | *Lux-*tagged PA strains NH57388A (mucoid) and MR299 (non-mucoid) isolated from patients with CF | Testing whether two newly-isolated phages at a multiplicity of infection (MOI)^***^ 2 to 5 can clear mucoid and non-mucoid *lux*-tagged PA in two 24-h-old PA biofilms growing on a monolayer of cystic fibrosis bronchial epithelial cells. Biofilm clearing monitored by measuring luminescence | PA biofilm luminescence decreased by 2 log  Bacterial cell numbers decreased by 3 to 4 log, whereas phage titers increased by 2 log  A mix of two phages reduces the risk of resistant mucoid and non-mucoid PA colonies | None | The study tested a mix of only two phages | The study tested mucoid and non-mucoid *lux-*tagged PA  PA biofilm were grown on a bronchial epithelial cell monolayer from a patient with CF |
| 2- Alves, 2015 (United Kingdom) | Six newly-isolated phages: DL52, DL60, DL68 (*Myoviridae*), DL54, DL62 and DL64 (*Podoviridae*) | Samples from sewage and flood water | PAO1 strain isolated from a patient’s wound | Testing the antimicrobial efficacy of six novel phages (singly and in cocktail formulation; MOI 0.1, 1, 10) against planktonic PAO1 culture, and in static and dynamic ﬂow bioﬁlm models | In planktonic PAO1 culture, for all single phage suspension inoculated, PAO1 regrew in 8-13 hours, whereas the phage cocktail inhibited PAO1 growth in bacterial culture  In planktonic PAO1 culture, phage cocktails exhibit a more positive and rapid effect in eliminating the bacterial load of broth cultures than for biofilms  Under static conditions, at 4 hours after phage addition the PAO1 biomass decreased (by 80% for MOI 1, and more than 95% for MOI 10) (P < 0.05). At 48 h, although at 24 h the biofilm biomass was still lower than the control biomass for both MOI cases (MOI 10, P = 0.015; MOI 1, P = 0.003), the bioﬁlm biomass measured was greater than the bioﬁlm biomass in controls  Under dynamic conditions, the PAO1 biomass bioﬁlm decreased at the 24-h endpoint, and the decrease became more evident at 48 h | None | The phage cocktail was tested against PAO1 strains and not against PA strains from patients with CF | Phage presence and active replication in PAO1 biofilm were checked under static flow conditions, and to mimic a more realistic infection situation, under dynamic flow conditions |

| 3- Betts, 2013 (France) | PEV2, LUZ7, LKD16 (*Podoviridae*) 14/1 (*Myoviridae*) | Environmental water samples and sewage | | PAO1 strain isolated from a patient’s wound | Training of 4 phages (improvement in phage activity through 6 serial passages on an ancestral PAO1 strain) | Less infectious phage increased their lytic activity from 80-85% to 100%  First evidence of bacteria evolving resistance, during phage training, to ancestor phage and cross-resistance to foreign phages | | Rising of resistant bacterial strains | Bacterial resistance was evaluated only after the first passage  Phages not tested against CF clinical strains | Evaluation of bacterial resistance and cross-resistance caused by phage treatment |
| --- | --- | --- | --- | --- | --- | --- | --- | --- | --- | --- |
| 4- Coulter, 2014 (Texas, USA) | PB-1 (*Myoviridae*) | A PB-1 sample from the American Type Culture Collection (ATCC) 15692-B3 | | PAO1 strain isolated from a patient’s wound | Tests evaluating the emergence of phage**-** and antibiotic-resistant cells, and biofilm survival giving a phage infection (MOI of 0.01) and tobramycin (0.5 µg/mL) simultaneously | Tobramycin and PB-1 combined on PA biofilms was just as effective as tobramycin alone in decreasing biofilm mass  During combined treatment tobramycin-resistant cells decreased by 60% and PB-1-resistant cells by 99% | | None | Phage and tobramycin combination not tested against CF clinical strains  Tobramycin inhibited protein synthesis, thus interfering with phage production | Predefined tobramycin and phage concentrations are useful to assess potential phage-antibiotic interactions |
| 5- Danis-Wlodarczyk, 2015 (Poland) | Two newly-isolated KT28 and KTN6 (*Myoviridae*) | Environmental water samples from irrigated fields | PAO1 and PA0038 strains isolated from a patient’s wound, and PA708 isolated from a patient with CF | | Tests evaluating the efficacy of different methods for biofilm eradication activity tested on peg-lid plate assay and PET membrane surface. The efficacy of biofilm degradation was tested by measuring the survival colony forming units by crystal violet (CV) staining, and analyzing pyocyanin and pyoverdine secretion by spectrophotometry (SPM), and fluorometry (FM). Only for PAO1 strain growth medium diffusion through biofilm matrix was measured by laser interferometry (IFM) and goniometry (GM) analyses | CV staining to test PA biofilm biomass showed unstable results. To test biofilm eradication activity were performed colony forming unit (CFU) count and pyocyanin and pyoverdine secretion detected by SPM and FM. CFU showed a significantly reduction (by 70-90%) in clinical PA cell numbers in 24 h-72 h-old biofilm cultures, and. SPM and FM showed pyocyanin and pyoverdine reduction, thus yielding phage activity in all tested PA strains. Laser IFM and GM analyses in the PA01 strain showed that biofilm structure disruption products increased after phage application  No phage mutants appeared during co-existence with resistant host variants | | After active KT28 and KTN6 phage treatment for one day, more than 90% of persistent PA cells became insensitive to both phages, and cross-resistance developed | Not specified whether PA708 (from a CF patient) is early or chronic, nor if it is a mucoid or non-mucoid phenotype  MOI not specified | After testing biofilm biomass reduction by CV staining, SPM, FM, CFU count and laser IFM and GM confirmed phage potency to reduce PA biofilm eradication activity |
| 6- Danis-Wlodarczyk, 2016 (Poland)** | One newly-isolated KTN4 (*Myoviridae*) | Sewage from irrigated fields | PAO1 and non-CF 0038 PA strains isolated from a patient’s wound, and the small colony variant PA strain CF708 isolated from a patient with CF | | Test evaluating KNT4 antibacterial potential using: biofilm assays (CV staining, IFM and profilometry, PFM), analysis of pyocyanin and pyoverdine/pyochelin secretion to the medium by SPM and FM tests, gentamicin exclusion assay on an airway surface liquid (ASL) model on non-CF (NuLi-1) and CF (CuFi-1) epithelial cell lines  Test evaluating phage and colistin (100 μM) given simultaneously  Experiment evaluating whether KTN4 influences the number of bacteria invading epithelial cell lines | With a CV staining assay, colistin alone significantly reduced the 24 h PAO1 biofilm biomass, whereas intact phage particle treatment increased it  KTN4 phage significantly inhibited PAO1 pyocyanin and pyoverdine/pyochelin production (p < 0.005)  Combined phage and antibiotic treatment showed no synergistic effect in either biomass or dye determination assays  In both ASL models, phage~~s~~ showed a strong bactericidal effect against the three PA strains (4–7 log reduction of colony count). Unlike the CF708 isolate, PAO1 was significantly more susceptible to phage treatment in NuLi-1 cells than in CuFi-1 cells (p < 0.05)  Whereas in the NuLi-1 cell line, KTN4 left CFU counts for all PA invading strains significantly unchanged, KTN4 phage application was significantly effective in wild type PA strains internalization prevention in CuFi-1 cells (p < 0.05)  CF708 isolate was able to internalize with the CuFi-1 epithelium in a relatively short time after infection evading phage lytic activity | | In a crystal violet- binding assay phage treatment increased the biofilm biomass | Colistin caused cell death by destabilizing cell membrane, thus limiting phage propagation  Phage not tested against CF clinical strains other than a small colony variant strain  MOI not specified | The gentamicin exclusion assay on an ASL *in vitro* model is flexible, generates reproducible data with well-controlled and standardized conditions, mimicking the normal and CF lung environments. It also provides the basis for understanding host-pathogen interactions and is, as such, an important step towards experimental *in vivo* studies  Phage effect on biofilm analyzed with CV staining and several other tests  Use of an ASL model on non-CF and CF epithelial cell lines |
| 7- Essoh, 2013 (France) | Six Pyophage-derived phages: P1-14_pyo_ (*Myoviridae*) P1-15_pyo_, P8-13_pyo_, P2-10_pyo_, P3-20_pyo_, PTr60_pyo_ (*Podoviridae*)  Two newly-isolated phages: P1-14_Or01_ (*Podoviridae*) and P2-10_Ab01_ (*Myoviridae*) | Pyophage preparation obtained from the Eliava Institute (Tbilisi, Georgia), and other newly-isolated samples from sewage | Forty-seven PA strains from different clusters isolated from patients with CF | | Plaque assay to evaluate bacterial susceptibility to individual phages or to 3 different cocktails (6 Pyophage-derived phages, the two newly-isolated phages or 8 isolated phages combined) | | Efficient cocktails contain several phage genera, but it is difficult to prepare a phage cocktail that efficiently reduces all PA strains  Phages behave similarly alone, or in a cocktail  Phages lysed 70% of PA strains  The CRISPR-Cas system was absent in the 13 phage-resistant strains | The combined Pyophage preparation or each newly-isolated phage individually failed to lyse 13 of the 47 PA strains | Bacteria sensitivity and phage host range determined by phage spotting on a lawn of stationary growing bacteria. This metabolic bacterial status could reduce phage lytic activity as others have shown (Abedon and Yin, 2009; Middelboe, 2000; Sillankorva et al., 2004) | Tests evaluating the presence of the resistance mechanism CRISPR-Cas in the 13 phage-resistant strains |
| 8- Friman, 2016, (United Kingdom) | 14/1, phiKZ, PT7 (*Myoviridae*) and PNM (*Podoviridae*) | Sewage and environmental (lake, river) water samples | Ten PA strains isolated from patients with CF (five with intermittent, and five with chronic infections) | | Evaluate whether pre-adapting phages to bacteria through *in vitro* pre-adaptation decreases the rate at which bacteria subsequently evolve resistance to them | | Trained (evolved) phages caused a greater reduction in PA bacterial densities than ancestral phages, and this difference was especially clear with chronic isolates  Phage therapy could work more uniformly across clinical bacterial isolates that share the same initial origin (e.g. certain environmental reservoir), or parallel evolutionary history in a similar within-host environment (e.g. lung)  Although during *in vitro* phage bacterial coevolution all bacteria evolved some resistance to ancestral phages, only 50% of bacterial strains became resistant to trained phages. Phage selection, measured in the absence of phages, led to decreased bacterial growth with greater and more consistent growth cost (fitness cost) for chronic than for intermittent isolates | At the end of the *in vitro* phage-PA co-evolution (5-days), PA became resistant to ancestral and trained phages | Limited number of PA strains used despite the considerable existing strain variation within and between individual patients with CF | Estimating the fitness cost for phage-resistant bacteria, especially comparing intermittent and chronic PA infections  Evaluating bacterial resistance to ancestral and trained phages when phage-PA co-evolution ends |

| 9- Garbe, 2010 (Germany) | One newly-isolated JG024 (*Myoviridae*) | Sample from sewage | PAO1, PA mucA mutant strain (mucoid variant of PAO1) and mucoid BT73 strain (isolated from a patient with CF) | Tests evaluating whether JG024 can infect 3 PA strains (PAO1, mucA and BT73) mimicking simulated CF lung conditions obtained by means of an artificial sputum medium (ASM)  Experiments evaluating how alginate influences phage infection efficiency | JG024 infecting PA01 in an ASM showed a 50-fold lower concentration of phage particles, indicating a reduced JG024 phage infection efficiency with this model  The PA mucA mutant PAO1 strain infected by JG024 determined a 1.6-fold phage number reduction in the ASM, and an overall approximately 10-fold greater reduction in phage particles than did PAO1  In an ASM, the BT73 CF mucoid strain infected by phage JG024 *in vitro* showed lower susceptibility than PAO1 and PA mucA mutant strains  The BT73 CF mucoid strain infected by JG024 reduced phage numbers 1.9-fold. This mucoid strain is less susceptible to JG024 lysis than the PAO1strain and produced about 10-fold fewer phage particles  The presence of alginate in Luria Bertani (LB) broth medium reduced phage multiplication from 20-fold (concentration of alginate equal to 50 to 200 μg/ml) to 2,800-fold (concentration of alginate equal to1 mg/ml) | None | Phage activity in ASM was tested against a single CF strain  The authors failed to specify whether the BT73 CF mucoid strain had been isolated from a CF patient with a chronic PA infection  Lack of information on possible phage-receptor changes in mucoid-strain cell membranes | Tests using two mucoid PA strain hosts and adding alginate in an LB broth provide evidence that alginate overproduction influences phage infection efficiency |
| --- | --- | --- | --- | --- | --- | --- | --- | --- |

| 10- Hall, 2012 (United Kingdom)^****^ | 14/1, phiKZ, PT7 (*Myoviridae*) and PNM (*Podoviridae*) | Sewage and environmental (lake, river) water samples | PAO1 strain isolated from a patient’s wound | Determine the best way (simultaneously or sequentially) to administer phage combinations at MOI 1 | Multi-phage therapy was more successful than single-phage therapy for reducing bacterial densities and caused no significant increase in the frequency of multi-resistance. Simultaneous application was consistently equal or superior to sequential application for reducing bacterial population density, estimated by optical density measurements (ODM)  Phage-resistant bacteria emerged in all experimental treatments and incurred significant fitness costs, expressed as reduced growth rate in the absence of phages. No difference (on average) observed for ability to minimize resistance | Emergence of phage-resistant bacteria | Phages not tested against CF clinical strains  Bacterial population densities estimated by ODM probably altered if genotypes resistant to phages overproduce alginate or extracellular polymeric substance | Evaluation of fitness costs in phage-resistant bacteria |
| --- | --- | --- | --- | --- | --- | --- | --- | --- |

| 11- Hanlon, 2001 (United Kingdom) | F116 (*Podoviridae*) and GL1 (NA) | F116 was obtained from the Welsh School of Pharmacy, University of Wales, United Kingdom, and GL1 from the School of Pharmaceutical Sciences, University of Nottingham, United Kingdom | The non-CF PA NCIMB 10548 and a PA mucoid strain from a patient with CF | Evaluation of phage ability to diffuse through PA NCIMB 10548 biofilms grown on discs of poly (methyl) methacrylate incubated at 37°C for up to 20 days. Phage suspensions of MOI 100 or 1000, were used to infect both intact biofilms and re-suspended in 5-, 10-, 15-, and 20-day-old biofilm cells and the mixtures were incubated at 37°C for 24 h. When exposure ended, the viable bacteria remaining in the biofilms were quantified  Investigation of phage ability to penetrate alginate gels containing different concentrations of commercial and purified CF alginate, through incubation with 2 x 10^10^ purified phage per ml for 24 h at 37°C. After incubation, the samples and controls were analyzed with a Carrimed-controlled stress rheometer. The experiment was repeated by using concentrations of 10^10^ and 10^12^ phage per ml, and samples were taken for analysis at intervals up to 4 h  To obtain degradation evidence, phage-treated and untreated commercial and purified CF alginate samples, were subjected to gel filtration chromatography | Exposure of biofilm cells to phage for 24 h showed that the number of viable bacteria in a biofilm diminished up to 99% despite the presence of exopolysaccharide. This reduction depended on the initial phage ratio to cells (1-log reduction with MOI 100, 2-log reduction with MOI 1000) and on whether the cells were attached to the substrate (2-log reduction with MO 100 and 3-log reduction with MOI 1000)  Younger biofilms seemed no more sensitive to phages than 20-day-old PA biofilms. PA biofilms of all ages resulted in the same log reduction factors for all treatments  Phage-treated sample viscosities diminished by up to 40% compared with those of controls incubated in the absence of phage in a time-and concentration-dependent way  Even at the highest concentration of alginate used (12% weight/volume), phage could penetrate during prolonged incubation. At the lower concentrations of commercial alginate and in the presence of purified CF PA exopolysaccharide, phage diffusion was rapid  The results of gel filtration chromatography demonstrated that phage-treated alginate had a lower molecular weight than untreated alginate, owing to phage enzymatic degradation  Reduction in viscosity appeared highly specific because phages targeting other bacterial species had no effect compared with controls | Cells grown planktonically in liquid cultures exhibited log reduction factors greater than 5, suggesting that the re-suspended biofilm-derived cells still had increased resistance to treatment | Phage activity against old biofilm tested using a laboratory PA strain instead of a CF strain.  F116 is a lysogenic not a lytic phage | Phage activity tested against 20-day-old PA biofilm  Evaluation of phage ability to diffuse throughout alginate purified from a mucoid PA strain from a patient with CF |
| --- | --- | --- | --- | --- | --- | --- | --- | --- |

| 12- Henry, 2013 (France)** | Nine newly-isolated phages: PAK_P1, PAK_P2, PAK_P3, PAK_P4, PAK_P5 (*Myoviridae*) hosted on the PAK strain; PhiKZ (*Myoviridae*), and LUZ19 (*Podoviridae*) amplified on PAO1; CHA_P1 hosted on the PA CHA strain; LBL3 (*Myoviridae*) hosted on the Aa245 strain | | Environmental sources | | The laboratory PAK, PAK-lumi (PAK bioluminescent version) and PAO1 strains, the multidrug-resistant-mucoid PA CHA strain isolated from a patient with CF and the Aa245 strain isolated form burn wound | | Efficiency of plating (EOP)**^†^** test assessed on both the PAK-lumi strain and the original hosts for each phage using the plaque assay method  Evaluation, in a 96-well plate reader, of lysis kinetics for each phage (MOI 0.001) in liquid LB medium using the PAK-lumi strain | Predicting *in vivo* efficacy of therapeutic phages against PA infections by testing *in vitro* EOP (good correlation between *in vitro* and *in vivo* results). EOP was set to 1 for the PAK_Px phages and was found to be 0.8 for LBL3 and 0.2 for LUZ19. PhiKZ had an EOP of 1.2, as more plaques were observed on the PAK-lumi strain than on the strain used for its propagation, PAO1. CHA_P1 phage was able to infect the PAK-lumi strain with an EOP of 0.5  In the lysis kinetics evaluation, after incubation for about 70 min the optical density (OD) decreased for all the PAK_Px phages. Two clusters were identified within this group, the first consisting of PAK_P1, PAK_P2, and PAK_P4, for which the OD value reached 0.04 before being affected by bacterial lysis, and the second consisting of PAK_P3 and PAK_P5, for which the OD value reached 0.055. The time lag to a decrease in the OD was the greatest for LUZ19 (about 270 min), suggesting that this phage induced lysis more slowly whereas the other phages induced lysis slightly more rapidly (PhiKZ 210 min and LBL3 180 min). The lysis kinetics for CHA_P1 were similar to those for the PAK_Px phages, although lysis was slightly less rapid | None | The experiments to test phage activity by EOP were conducted using as the phage host the laboratory PAK-lumi strain and not a CF strain | EOP test used to define the lytic activity of each phage and compare it with the phage activity observed on PA hosts |
| --- | --- | --- | --- | --- | --- | --- | --- | --- | --- | --- | --- |
| 13- Larché, 2012 (France) | Three newly-isolated phages: A, B, C (NA) | | Samples from a Parisian wastewater system | | Forty-four multi-drug-resistant (MDR) and extensively-drug-resistant (XDR) PA strains belonging to different clonal complexes from patients with unspecified pathologies | | Plaque assay to evaluate PA susceptibility to phages individually or combined in a cocktail | Forty-two PA strains (95.4%) displayed high susceptibility to at least one phage, as well as to the cocktail  In isolates from two clones. genotype patterns correlated with susceptibility patterns  Phage therapy could be an alternative or complementary treatment to antibiotics for MDR and XDR PA infected patients | Two isolates belonging to the same lineage (representing alone the 50% MDR isolates), showed resistance to all three phages, both individually and in cocktail | Because the authors incompletely described DNA analysis whether phages are lytic remained unknown  PA susceptibility tested by spotting phage on a lawn of bacteria  PA strain resistance evaluated from different laboratories from different countries, thus reducing generalizability of the results | Even though unspecified, PA origin investigators tested phages against MDR and XDR PA strains |
| 14- Lehman, 2016 (United Kingdom)^**‡^ | | Four newly-isolated phages combined in the cocktail AB-PA01 (NA) | | Environmental sources in Australia and the UK | Three-hundred sixty-nine PA strains from patients with CF, and 60 PA strains from non-CF patients, collected from 2007 to 2015. PA isolates included both antibiotic susceptible/ resistant and mucoid/non-mucoid strains | | Plaque assay to evaluate bacterial susceptibility to phages combined in a cocktail  The number of isolates targeted by ≥2 phages considered an important selection criteria  The experiment checked individual PA responses to phages | The phage mix infected both antibiotic susceptible or resistant and mucoid or non-mucoid CF PA strains, showing that AB-PA01 has a broad range of activity. Of a total 87.8% CF PA isolates, sensitive to the AB-PAO1 cocktail, 93.2% of PA strains were lysed by at least 2 phages in the cocktail. Of a total 87.2% of non-CF PA isolates sensitive to the AB-PAO1 cocktail, 91.4% of PA strains were lysed by at least 2 phages in the cocktail | A total 12.2% CF PA strains were insensitive to the phage cocktail | Bacterial susceptibility tested only by spotting phage on a lawn of bacteria | Use of CF antibiotic susceptible or resistant and mucoid or non-mucoid PA strains |
| 15- Lim, 2016 (Republic of Singapore) | | PB1 (*Myoviridae*) | | NA | PAO1 strain isolated from a patient’s wound | | Isolation of small colony variants from the surviving population of wild-type PAO1 exposed for 24h to PB1 phage and evaluation of their characteristics and susceptibility to phage | The small colony variant isolates were found to be resistant to further PB1 infection and displayed several phenotypic changes (were less competent at attaching to surfaces in the biofilm formation assay, produced lower amounts of the virulence factors elastase and pyocyanin, and twitched less than the wild-type PAO1). The lower expression of pyocyanin and elastase in small colony variants may indicate that the *las* quorum sensing system is deficient in these strains  Transcriptomic studies disclosed up-regulation of genes involved in O-specific antigen (OSA) biosynthesis, suggesting that surface moiety regulation may account for phage resistance  Changes to the OSA could play a role in preventing binding of phage particles and initializing the infection  Whole genome sequencing shows single nucleotide variations predominantly in the Pf1 prophage region  No difference observed in gentamicin susceptibility but an increase in ciprofloxacin susceptibility in PAO1 small colony variants | None | Experiment conducted using PAO1 and no CF strains | Phage resistant small colony variant PAO1 mechanisms investigated by the means of phenotypic assays, DNA microarrays and whole-genome sequencing |
| 16- Olszak, 2015 (Poland)** | | 28 newly-isolated phages, 2 of which were characterized: PA5oct and KT28 (*Myoviridae*) | | Natural wastewater treatment plant (irrigated fields) | | A total 121 PA strains (including 29 mucoid strains) isolated from patients with CF with diverse degrees of virulence, PAO1 and a clinical non-CF strain | Correlation between phage activity and PA strain features (mucoid, twitching motility, biofilm production, and biochemical composition) evaluated using the spot test | Features influencing phage efficacy are slow growth rate, low biofilm production, reduced twitching motility and chemical composition. Of the 28 phages tested, at least 3 lysed 93.6% of PA strains, whereas 6.4% of PA strains were completely insensitive to phage activity | None | Phage activity tested only by spot test | Determining which PA host features influence phage activity in CF and non-CF PA strains isolated from colonized patients |
| 17- Pires, 2011 (Portugal) | | Newly-isolated: phiIBB-PAA2, phiIBB-PAC23, phiIBB-PACL12, phiIBB-PAP21 (*Podoviridae*) | | Two hospital effluents | | PAO1 strain isolated from a patient’s wound and an ATCC 10145 strain of unspecified origin | Isolation of new phages and evaluation of their ability to control planktonic cultures and 24 h-old biofilms (MOI 1) | After treatment for 24 h, phiIBB-PAA2 reduced the biomass by 3 log in ATCC 10145 biofilm  Conversely, the phiIBB-PAP21phage induced a 3-log PAO1 biofilm biomass reduction in the first 6 h, followed by bacterial regrowth  Phages can be equally efficient towards stationary and exponential phase cells | Phages isolated from clinical PA strains have a reduced host range  Despite broad lytic spectra, phage can fail to infect planktonic cultures  Planktonic culture**s** and biofilm can become phage-resistant after the first 6-10 hours  Higher amounts of the phiIBB-PAA2 than the phiIBB-PaP21 phage were observed entrapped in the biofilms after 2-hour infection | Phages not tested against CF clinical strains | Checking phage presence in biofilm demonstrated their presence and their active replication during treatment |

| 18- Saussereau, 2014 (France) | PAK_P1, PAK_P2, PAK_P3, PAK_P4, PAK_P5, P3_CHA, CHA_P1, phiKZ, LBL3 (*Myoviridae*) and LUZ19 (*Podoviridae*) | Environmental sources | Twenty small or large PA-colonies, including mucoid and non-mucoid PA strains, from four aliquots collected from 48 sputum samples from patients with CF in three French hospitals | Sputum used to evaluate PA and phage counts before and after addition of the phage cocktail over 6 h  Tests evaluating the sensitivity of individual PA colonies to each phage in the cocktail. As a standard procedure, the authors specify that **i**solated plaques, confluent plaques or entirely clear areas were considered to indicate that the bacteria were susceptible to the phage tested, whereas the absence of plaques indicated fully resistant strains | Phages PAK_P5, LBL3, PAK_P3 and PAK_P4 were the most efficient, whereas phages CHA_P1 and PhiKZ were the least efficient. The total number of PA increased during the incubation period in the absence of phages (p < 0.001). Despite this growth, the addition of phages significantly decreased the number of bacteria present (p 0.024) and in 86.4% of the samples the number of phages increased over the threshold value  For isolating new phages from environmental sources, 5 PA colonies were selected, all of which were resistant to all 10 phages. Of these new phages, three chosen at random, infected all five PA colonies, demonstrating that these colonies were susceptible to phage activity  None of the clinical criteria retrospectively tested in patients with CF were associated, either positively or negatively, with phage efficacy. Hence the microenvironment in the lungs of patients with CF has no influence on phage efficacy (patient-independent efficacy) | None | Inadequate information on phage lytic activity evaluated on mucoid and non-mucoid PA strains collected from CF patients’ sputum in a selective medium | Evidence suggesting that new phage cocktails should be formulated for each patient with CF (personalized approach) by testing a set of newly-isolated phages against a large panel of resistant PA strains isolated from pulmonary chronic infections in patients with CF |
| --- | --- | --- | --- | --- | --- | --- | --- | --- |

| 19- Torres-Barceló, 2014 (France) | LUZ7 (*Podoviridae*) | Hospital sewage samples | PAO1 isolated from a patient’s wound | Experiments testing changes in PAO1 after independent and combined treatments with a phage and streptomycin (100 or 240 µg/mL).  The phage and streptomycin were tested at different time points: simultaneously, at 12 h and at 24 h after phage administration | Single treatments strongly reduced PAO1density over the first 24 h. At the 70-h endpoint, the treated samples reached untreated control levels  The combined treatment of a phage at an unspecified MOI, and streptomycin caused a significantly stronger reduction in PA density than either single treatment, and strongly suppressed final densities. PA density, antibiotic resistance and increase in PA phage resistance tended to be minimal when streptomycin was added to phages with a 12 h delay (peak phage efficacy)  Streptomycin dose (100 vs. 240 µg/mL) had no significant effect on final density  Combined treatment provided no evidence for a trade-off between antibiotic resistance and phage resistance. Higher phage resistant levels were associated with higher antibiotic resistance | None | Phage and streptomycin combination not tested against CF clinical strains  MOI not specified | Independent and synergistic effect of combined phage and streptomycin treatment  Predefining sub-lethal and minimum inhibitory concentration (MIC) streptomycin for PAO1  Detecting the optimal window for antibiotic administration (i.e. 12 h after adding phages)  PA exposed to the combined treatment for 70 h |
| --- | --- | --- | --- | --- | --- | --- | --- | --- |

| 20- Uchiyama, 2016 (Japan) | Newly-isolated KPP22 (*Myoviridae*) | NA | PAO1 isolated from a patient’s wound | Tests evaluating short-term antagonistic evolution of bacteria and phages by studying phage-resistant PA clones, and mutant phages with recovered infectivity | All three KPP22-resistant PAO1 clones were deficient for the O5 antigen, and had a common nonsense mutation in the wzy gene. All the KPP22 mutant phage genomes showed the same four missense mutations in the open reading frames orf060, orf065, and orf086.  EOPs of ancestral KPP22 phage on the KPP22-PAO1 resistant clones were significantly lower (ca. 10^−4^ to 10^−5^ times) than that on the laboratory PAO1 host strain. In contrast, the EOPs of the three mutant KPP22 phages on the KPP22-resistant PAO1 clones showed the same efficacy expressed against the laboratory PAO1 host strain  Bacterial resistance to phage is caused by significant decreases in phage adsorption, whereas improved KPP22 mutant phage infectivity is caused by significant increases in phage adsorption | None | Phage not tested against CF clinical strains | Genetic analysis including resistant PAO1 clones and mutant phages |
| --- | --- | --- | --- | --- | --- | --- | --- | --- |

*Abbreviations: CF, cystic fibrosis; NA, data not available; ^*^taxonomy in accordance with the International Committee on Taxonomy of Viruses. Available at:* [*https://talk.ictvonline.org/taxonomy/*](https://talk.ictvonline.org/taxonomy/); *^***^MOI, multiplicity of infection = the ratio between the plaque-forming units (PFU) and the colony-forming units (CFU); ^***^results in vivo in* ***Additional file 2****; ^****^the in vivo results from this study were excluded because they exclusively referred to PAO1 infecting a non-pulmonary animal model; ^†^EOP, efficiency of plating = the ratio between the average PFU on target bacteria and average PFU on host bacteria; ^‡^poster presented at the European Congress of Clinical Microbiology and Infectious Diseases 2016. No published results.*
