# Supplementary material for: Challenges and Promises for Planning Future Clinical Research Into Bacteriophage Therapy Against Pseudomonas aeruginosa in Cystic Fibrosis. An Argumentative Review
Source: Front Microbiol. 2018 May 4;9:775. doi: 10.3389/fmicb.2018.00775 (PMC5945972; doi:10.3389/fmicb.2018.00775)
Supplement: Supplementary file 2 [file Data_Sheet_2.docx]

| **Additional file 2 \| Line of arguments from 9 studies included in the review investigating *in vivo* lytic bacteriophage (phage) effects against laboratory *Pseudomonas aeruginosa* (PA) strains or non- cystic fibrosis (CF) strains or PA strain hosts isolated from patients’ wounds, patients with diseases other than CF, and patients with CF in pulmonary, and non-pulmonary host models (PA-infected animal models) (studies reported alphabetically according to the first authors’ surnames).** | | | | | | | | | | |
| --- | --- | --- | --- | --- | --- | --- | --- | --- | --- | --- |
| **First author, year (Country)** | **Phage taxonomy (family)*** | **Phage sources** | **PA strain hosts** | **Control group treatments** | **PA infection in animal host models** | **Procedure** | **Results** | **Problems reported on safety and efficacy** | **Limitations** | **Strengths** |
| 1- Alemayehu, 2012 (Ireland)** | Two newly-isolated φNH-4 (*Myoviridae*) and φMR299-2 (*Podoviridae*) | Sample from sewage | *Lux*-tagged PA strains NH57388A (mucoid) and MR299 (non-mucoid) isolated from patients with CF | Infected control group received PBS instead of phage mix | Mice (8-week-old BALB/c female) in intervention and one control group infected with *Lux*-tagged PA strains NH57388A or MR299 strains causing acute lung infection | Evaluating whether phage can kill PA in situ in the lungs of infected 8-week-old female BALB/c mice. Mice infected intra-nasally with *lux*-tagged Pseudomonas NH57388A or MR299 were treated two hours later with a phage mix suspension (MOI^***^ 10), given intra-nasally. Bacterial clearing monitored by measuring luminescence | The two phage mix is effective in killing *lux*-tagged PA in the lungs of infected mice  During the 6-hour experimental procedure, phage treatment prevented growth and reduced the bacterial load to a non-detectable level | None | Lack of a negative control group  The study tested only a two-phage cocktail  Phage host range evaluated only by plaque assay, and not by assessing efficiency of plating (EOP)^****^ | The study tested mucoid and non-mucoid *lux*-tagged PA |

| 2- Beeton, 2015 (United Kingdom) | Newly-isolated DL52, DL60, DL68 (*Myoviridae*). DL54, DL62, DL64 (*Podoviridae*) | Sewage and flood water samples | PAO1, PA45291 (isolated from bacteremia) and BC09007 (isolated from a patient with CF) | Three control groups, two negative (one of PA uninfected-PBS injected larvae, to assess the impact of any negative effect from the injection process; and one of PA uninfected phage-treated larvae to assess the toxicity of the phage cocktail), and one positive (PA infected larvae treated with PBS solution) | Wax moth larvae from *Galleria mellonella* in the intervention and in the positive control group were infected with PAO1 or clinical strains PA45291 and BC09007. Uninfected PA larvae in the negative control group received PBS | Evaluating whether a cocktail of 6 phages can treat or prevent infection caused by 10 or 100 PAO1 cells delivered into the larval haemolymph. Two different approaches tested: in the curative model, phage suspensions are delivered 2 h post-infection at a MOI 0.1, 1 or 10, whereas in the preventive model phage suspensions are delivered 2 h pre-infection at a MOI 0.1, 1, 10, 100. Larvae were incubated at 37°C and mortality was observed over 48 h. Validation of the preventive model with two clinical strains. Larvae, pre-treated with phage at a MOI 10 at 2 h pre-infection, were infected with 10 cells of PA45291 or BC09007 | In the curative model administering phages prolonged *Galleria mellonella* survival in a dose-dependent manner (the higher the MOI the better the result). At 24 h post-infection, phages kept bacterial cell numbers 1000-fold lower in phage-treated larval haemolymph than in the non-treated group (positive controls); all MOI achieved 0% survival at 30 h. In the preventive model, survival at 24 h ranged from 80% in larvae infected with 100 cells and given at MOI 100 to 35% in those given at MOI 0.1. Survival ranged from 90% to 60% in larvae infected with 10 cells and given at MOIs 100 and 1 respectively. Infection with the acute isolate PA45291 resulted in rapid *Galleria mellonella* mortality within 24 h, with 85% survival (phages given at MOI 10). The BC09007 CF isolate was less virulent at 24 h than the bacteremic and PAO1 strains, it achieved 100% mortality at 40 h  The investigators suggest that continual PAO1 survival in the presence of a high phage titre could be caused by intracellular localization of PA, having ruled out phage resistance developing within the larvae  *Galleria mellonella* is a simple, robust and cost-effective model for initial *in vivo* examination of PA targeted phage therapy | Phage-treated Phage-treated *Galleria mellonella* infected by PAO1 died at 30 h post-infection and larvae infected by clinical strains PA45291 and BC09007 eventually succumbed at 40 h | The authors failed to provide information on the reason why they use phage suspensions at a MOI 100 only for the preventive model (probably owing to experimental risks including killing the larvae) | To rule out PAO1 possible phage resistance developing during *in vivo* infection, re-isolated PAO1 were plaque assayed to confirm susceptibility |
| --- | --- | --- | --- | --- | --- | --- | --- | --- | --- | --- |

| 3- Danis-Wlodarczyk, 2016 (Poland)** | Newly-isolated KTN4 (*Myoviridae*) | Sewage from irrigated fields | PA O1 and non-CF0038 strains isolated from a patient’s wound and the small colony variant CF708 strain isolated from a patient with CF | The controls consisted of PA uninfected larvae receiving phage lysate only (negative), and larvae infected with bacterial lethal dose (positive) | Wax moth larvae from *Galleria mellonella* infected with PAO1, non-CF0038 or CF708 strains in the intervention and in the positive control group, causing lethal systemic infection. Uninfected PA larvae in the negative control group received only phage lysate | Larvae infected by injecting the ventral side of the last pseudopod pair with a lethal dose of bacterial cells (10 CFU for PAO1 and non-CF0038 strains and 10^6^ CFU of CF708 isolate per larvae). Larvae were incubated for 96/120 hours at 37 °C. The phage lysate was injected at MOI 100. The results were read at 18, 24, 36, 48, 72 and 96/120 hours post injection and were expressed as the percentage survival rate assessed by macroscopic appearance | The KTN4 phage has an antibacterial strain-dependent efficacy against clinical isolates. The KTN4 phage application significantly increased the larval survival rate from lethal PAO1 infection, with 90% of alive caterpillars 36 h post injection. The protective activity of the phage was less efficient against non-CF0038 propagation than against PAO1, saving only 20% of larvae at a specific time. The antibacterial activity of applied phage against the CF708 isolate became evident only when the experiment ended (fourth/fifth day) with a higher survival rate for treated larvae than for untreated controls (68% vs 50%) | None reported | Because it grows slowly and leads to slow biofilm formation, low type IV pili expression, and lower virulence than PA O1 and non-CF0038, the small colony variant CF 708 strain is not a representative PA strain colonizing patients with CF | Incubation prolonged up to 120 hours |
| --- | --- | --- | --- | --- | --- | --- | --- | --- | --- | --- |

| 4- Debarbieux, 2010 (France) | Newly-isolated PAK-P1 (*Myoviridae*) | Sewage water | Biolumi nescent non-mucoid non-CF PAK strain | For curative treatment: PA uninfected phage-treated mice (negative), infected-PBS-treated mice (positive). For the preventive treatment, infected PBS-pretreated mice (positive) | Mice (8-week-old Balb/c males) infected with bioluminescent non-mucoid non-CF PAK strain in the intervention and in the positive control group, causing acute lung infection. PA uninfected mice in the negative control group received only the phage | Evaluation of the phage efficacy to treat and prevent acute lung infection. Bacteria and phages were intra-nasally instilled. In curative experiments, 2 h after bacterial instillation the bioluminescence was recorded and a MOI 10 of phages applied intra-nasally  In preventive experiments, 24 h before infection the animals received intra-nasally a MOI 10 of phages. Mice monitored for 16 days. BALs analyzed at the indicated time points to evaluate bacterial load, phage amounts and inflammatory marker levels, namely tumor necrosis factor α and interleukin 6  Bacterial clearing monitored by measuring luminescence | The respiratory tract from the upper parts to the lower parts could be treated with phage. Phages are not rapidly eliminated in the lungs. Phage efficacy in PA infected mice is dose and time-dependent. For curative treatment, at the 6 h time point (i.e. 4 h after phage administration), the amount of light emitted from phage-treated mice was statistically significantly lower than the amount of light emitted from the non-treated mice, suggesting rapid killing of bacteria by phages. At 24 h after the start of infection, treated mice showed no or only weak spots of light, whereas non-treated mice were highly luminescent or dead. Accordingly, only 1,5 x 10^2^ bacteria/mL, together with 2 x 10^7^ phages /mL, was recovered from phage-treated mice BALs at 24 h after the start of infection; at the same time, the levels of TNF-α and IL-6 (induced by a bacterial challenge) were statistically significantly reduced in the phage-treated group in comparison with the untreated group. At 48 h after infection, IL-6 and TNF-α levels returned to the baseline values in the phage-treated group  The safety was evaluated on phage treatment at MOI 100 and mice monitored for 10 days. Phage treatment was harmless to animals  In the preventive treatment, 2 h after bacterial inoculation, the amount of light emitted was ∼ 5 times lower in phage-pretreated mice than in control mice | When the experiment ended, 100% of phage-pretreated animals survived, whereas 100% of untreated animals died within 2 days | No CF PA strains were tested  The authors suggest that the presence of PAK-P1 in BALs of uninfected phage-treated mice, 24 h after phage administration, provides evidence that phages multiplied inside the lungs of infected and uninfected phage-treated animals. However, theoretically, the absence of a bacterial host in the PA uninfected animals impedes phage replication, hence viral load showed a 2 log decrease in 24 h | Use of biolumi nescent non-CF PAK strain. Evaluated the safety of phage treatment in a group of mice treated with MOI 100 and monitored for 10 days. Determination of the maximum possible delay (2 h) of phage treatment to maintain an animal survival rate of 100%. Evidence shows the need for using harmlessly active phage to cure non-mucoid non-CF PAK-infected mice. Measuring the level of lactate dehydrogenase (an enzyme released when cell integrity is damaged) present in BALs 6 h after the infection to identify the time in which bacteria multiply fastest, and, consequently, bacterial susceptibility to phage infection is highest |
| --- | --- | --- | --- | --- | --- | --- | --- | --- | --- | --- |

| 5- Henry, 2013 (France)** | Nine newly-isolated phages: PAK_P1, PAK_P2, PAK_P3, PAK_P4, PAK_P5 (*Myoviridae*) hosted on the PAK strain; PhiKZ (*Myoviridae*), and LUZ19 (*Podoviridae*) amplified on PAO1; CHA_P1 hosted on the PA CHA strain; LBL3 (*Myoviridae*) hosted on the Aa245 strain | Environmental sources | A bio luminescent non-mucoid non-CF PAK strain (PAK-lumi) | Infected mice treated with PAK_P1 (MOI 0.1) or PBS | Mice (eight-week-old, BALB/c males) in the intervention and in the positive control group infected with a PAK-lumi strain causing acute lung infection. Uninfected PA mice in the negative control groups were PBS or phage treated | Experiment comparing phage activity in a set of nine different phages to find a good correlation between *in vitro* and *in vivo* activity. Mice were infected by intra-nasally instilling 1 x 10^7^ CFU of PAK-lumi. Two hours later, mice were treated by the same route with a phage solution (MOIs between 0.05 and 0.2). The survival, weight, fur and motility features were monitored daily for 13 days after infection. Luminescence was measured for all animals at 2, 4, 6, and 8 h postinfection and then once every 2 days over 13 days | Bioluminescence emitted during the first 8 h of infection could be used to anticipate the overall *in vivo* efficacy of a given phage by defining a predictive index from the ratio of the luminescence values obtained at 2 and 8 h postinfection. A comparison of the median values in each series between 4 and 6 h after infection (i.e. between 2 and 4 h after phage treatment) showed a decrease in luminescence in PAK-Px-phage-treated mice (this infected group showed the highest survival rates, between 75 and 100%). The optimal efficacy achieved with the five PAK-Px phages isolated on the PAK-lumi strain highlighed a possible effect of the bacterial host used for isolation on the efficacy of the treatment For the LBL3 phage, luminescence did not begin to decrease until 6 h post-infection (survival rates of 50%). For LUZ19, the amount of light emitted decreased between 4 and 8 h after infection (survival rates 37%), and for PhiKZ, the light emitted from the chest area increased steadily during the first 8 h after infection (survival rates 15%), as for the PBS group. *In vitro* and *in vivo* efficacy correlated for most of the phages studied. As an index to discriminate *in vivo* phage efficacy the authors chose a ratio value of 3 between the luminescence values obtained at 2 and 8 h post-infection. For ratio values below 3, phage treatment resulted in survival rates of at least 75%, whereas for ratio values above 3, phage treatment allowed no more than 50% of the mice to survive | The *in vitro* efficacy of each of the nine phages was compared to their *in vivo* efficacy in a lung infection model, in which bioluminescence was used for the real-time monitoring of infection, evidencing a good correlation between *in vitro* results and *in vivo* efficacy for seven phages  CHA_P1 phage (isolated on a clinical PA strain CHA) was unable to cure animals infected with the PAK-lumi strain, despite being genetically closely related to PAK_P3 and PAK_P5. In addition, an attempt to adapt this phage to the PAK strain failed | *In vitro* efficacy is not sufficient to ensure *in vivo* efficacy  The experiments testing phage activity by EOP conducted using the laboratory PAK-lumi strain and not a CF strain as the phage host | Use of a real-time imaging system, and a treatment strategy that could be adapted to humans in a context of active therapy (minimal dose and an effect relying on the amplification of the phage numbers on the site of the infection) |
| --- | --- | --- | --- | --- | --- | --- | --- | --- | --- | --- |

| 6- Lehman, 2016 (United Kingdom)**^†^ | Four newly-isolated phages combined in the cocktail AB-PA01 (not reported) | Environ mental sources in Australia and the UK | Three hundred and 69 PA strains from patients with CF and 60 PA strains from non-CF patients collected between 2007 to 2015. Isolates included both antibiotic susceptible/resistant and mucoid/non-mucoid strains | Unspecified non-CF or CF PA-infected mice treated with the phage diluent | PA-infected CD-1 immunocompetent female mice in the intervention and in the positive control groups | Demonstrate the efficacy of AB-PA01 in infected mice and in comparison with antibiotic treatment (meropenem). Mice infected with PA intranasally were treated with phage 2 hours later with three different phage concentrations (1.5 x 10^9^, 1.5 x 10^8^, or 1.5 x 10^7^ PFU per dose). A second identical dose was administered at 6 h post infetion. Meropenem (25 mg/kg) was injected subcutaneously at 2 h and 6 h post infection to a fourth group of mice. All mice were killed at 24 hours and the CFU/lung pair was determined | AB-PA01 administered by nebulization at three dose levels demonstrated efficacy similar to meropenem in a PA murine lung infection model  A non-significant trend suggests a possible dose-dependent effect  Authors reported also long-term phage stability | None reported in the poster | The PA strains used to infect mice were not specified.  No negative control group reported | Use of 369 PA strains from patients with CF  Use of antibiotic susceptible/resistant and mucoid/non-mucoid strains |
| --- | --- | --- | --- | --- | --- | --- | --- | --- | --- | --- |

| 7- Morello, 2011 (France) | P3-CHA derived and trained (optimized) from PAK-P3 (*Myoviridae*) | P3-CHA derived and trained from PAK-P3 isolated from the environment | Multidrug-resistant and mucoid PA CHA strain isolated from a patient with CF | Curative treatment involved PA CHA infected-PBS treated mice (positive control) and PA CHA uninfected heat-killed P3-CHA treated mice (negative control). Preventive phage treatment involved heat-killed P3-CHA pre-treated PA CHA infected mice (positive control) and P3-CHA pre-treated uninfected mice (negative control) | Mice (8 weeks old Balb/c males) in the intervention and in the positive control group infected with PA CHA causing acute lung infection. Mice in the negative control groups were PA CHA uninfected | Tests evaluating the efficacy of curative and preventive phage treatments for lung infections caused by the PA CHA using an in-vitro-trained P3-CHA phage. For curative treatment, mice given a lethal dose of the PA CHA (3x10^6^ CFU) 2 h earlier were given two different P3-CHA doses (MOI 10 and 100) intra-nasally (mimicking a nebulization treatment for humans) and followed up for 16 days to evaluate the survival rate. The amount of bacteria, phages, cytokines, and LDH was quantified in BAL 20 h after infection, and histological and immune-histochemical data were analyzed. For preventive treatment: two groups of mice, pre-treated four days earlier with two different P3-CHA phage doses (MOI 10 and 100) were infected with 3×10^6^ CFU of PA CHA , and 20 h after infection the amount of bacteria, phages, cytokines, and LDH was quantified in BAL, and histological and immune-histochemical data were analyzed | Optimizing *in vitro* a phage towards a CF multidrug resistant clinical strain improves its efficacy in curative and preventive experiments *in vivo.* For curative treatment, the MOI 100 P3-CHA phage exhibited higher ability to rescue infected mice, compared with same MOI of PAK-P3 (95% and 20% of survival rate after 16 days). Phage P3-CHA improved survival at both doses, and the high dose was associated with a greater rate of survival (95%), number of PA cells lower than in the untreated group and the number of phages ten times higher.  Cytokines and LDH concentrations were markedly lower in the phage-treated group and also lung damage, observed with histological analyses, was less severe than in the untreated animals. Immunohistochemistry detected few PA (entire cells or debris) in phage-treated animals, whereas PA were detected in macrophages, alveoli, and extracellular spaces of the lungs from untreated animals.  Curative phage treatment acted cooperatively with the mice immune response to eliminate acute lung infection caused by PA CHA. A four-day preventive treatment resulted in 100% survival. Bacterial counts were lower in P3-CHA pre-treated mice than in positive controls. Lung immuno-histochemistry showed a pattern similar to curative treatment.  Preventive phage treatment required non-heat-killed active phage | None reported | For curative treatment, phage concentration data reported in the table and in the results showed major discrepancies, thus determining possible reporting bias | Tests using a multidrug-resistant mucoid strain from a patient with CF  *In vitro* phage optimization in tests using an endotoxin-free phage solution to reduce the possibility of stimulating a host immune response that could mask the effects of phage treatment |
| --- | --- | --- | --- | --- | --- | --- | --- | --- | --- | --- |

| 8- Olszak, 2015 (Poland)** | 28 newly-isolated phages (only two characterized): PA5oct and KT28 (Myoviridae) | Natural wastewater treatment plant (irrigated fields) | PAO1, non-CF0038 strain and 4 clinical CF strains (CF217, CF708, CF532, CF832) with diverse degrees of virulence | Uninfected larvae, sham-infected larvae, larvae receiving phage lysate only (negative), PA infected-untreated larvae, and infected larvae treated with UV inactivated phages (positive) | Wax moth larvae from *Galleria mellonella* infected with six PA strains causing lethal systemic infection. Larvae in the intervention and positive control groups infected with various PA strains. PA uninfected larvae in the negative control group | *Galleria mellonella* larvae infected with six different PA strains and treated with single or selected phage cocktail preparations to evaluate their antibacterial activity. Bacteria and phages were administered to larvae by injection into the ventral side of the last pair of pseudopods. For assessing antibacterial activity in selected phages, larvae were injected with the bacterial suspension and, within 1 h, with a MOI 100 single or phage mix. After injection, the larvae were incubated for 72 h at 37 °C. The effects of infection were checked at 8, 24, 48, 72, and 96 h after injection by assessing survival and macroscopic appearance. The results were expressed as percentage survival rates | Considering both non-CF PA strains (PAO1, non-CF0038), PA5oct was the least effective whereas phage KT28 was the most potent, rescuing around 20% of larvae even 2 days after lethal dose application. The application of a single PA5oct or KT28 preparation to cure infection in the phage resistant strain CF217 was ineffective, whereas a mixture of both lysates in the final MOI 100 increased the survival rate by up to 30% at 24 h, and 20% at 48 h. The phage cocktail against non CF PA infected larvae induced lower antibacterial protective activity than the single phage application. Inoculating an inactivated phage cocktail, in larvae infected with highly virulent CF isolates (i.e biofilm-forming non-mucoid CF217) at the lethal dose (10 CFU) lower than the lethal dose used for the weakly virulent strains (CF708, CF532, CF832) (10^5^ vs 10 CFU) showed larvae survival at a phage MOI value of 100. For weakly virulent CF isolates the phage inoculum was also higher to keep the MOI value of 100, thus causing larvae mortality owing to toxic compounds released from bacterial lysis. Larval survival reflected phage lytic activity rather than host immune stimulation (a finding confirmed using an inactivated phage cocktail) | Phage failure in rescue larvae infected with the 3 weakly virulent CF isolates could depend on the toxic compounds released during massive bacterial cell lysis after phage propagation, thus causing greater mortality in treated than in untreated larvae | Phages chosen for cocktail preparations were not selected for different host receptor affinity.  Because the mixture was less effective in lysing PAO1 and non-CF0038 than single lytic phage injection, phages probably competed for bacterial receptors | Use of UV-inactivated phages to assess their ability to rescue infected larvae to determine whether the effects of phage therapy were associated with a nonspecific immune activation response  Use of different CF strains, with diverse degrees of virulence |
| --- | --- | --- | --- | --- | --- | --- | --- | --- | --- | --- |

| 9- Pabary, 2016 (United Kingdom) | Phage cocktail PA 24, PA 25, PA 7 (NA) | Environ mental sources | PAO1 and PA 12B-4973 (isolated from a patient with CF) | Infected-sodium magnesium buffer-treated mice (positive) | Mice (adult, BALB/c, female) infected with PAO1 or PA 12B-4973 causing acute lung infection. Mice in intervention and positive control groups infected with PAO1 or PA 12B-4973 strains | Tests evaluating the effects of a phage cocktail on lung bacterial load, systemic spread of infection, and pulmonary inflammation. Tests exploring the effects induced by a therapeutic cocktail using three different approaches. Phages were administered either simultaneously, 24 h post-infection, or 48 h pre-infection. In the simultaneous treatment, two strains were used to infect mice: the PAO1 at low and high doses, and the CF PA strain 12B-4973 at low dose. Mice were intranasally treated with a phage MOI 1000 (for the lowest PA dose) and phage MOI 100 (for the highest PA dose). In the delayed and prophylactic phage administration, mice were infected with a PAO1 strain at a high dose, and inoculated with phages at MOI 100  BAL fluids were obtained 24 h after phage administration (for simultaneous and delayed approaches) or after PAO1 infection (in the prophylactic approach) to analyze inflammatory cytokine level, and quantify bacterial cells. Spleens were dissected and homogenized for defining PA presence, as an expression of systemic infection spread, in non-quantified cultures | In the simultaneous approach, with low infective doses no difference in the infective burden was demonstrated, because control and treated mice were both capable of spontaneous clearance during 48 h, but BAL from phage-treated mice contained fewer neutrophils than BAL from control mice. When PAO1 was injected at higher infective doses, all phage-treated mice cleared PA infection at 24 h, whereas in control mice infection persisted. When phages were given at 24 h after PA infection, complete clearance was seen in 6/7 (86%) phage-treated mice, and the median CFU/ml was significantly lower than that in control mice, who all had PA positive BAL cultures. When phages were prophylactically given, 5/7 phage-pre-treated mice (71%) successfully cleared the infection, and those that did not had only low levels of bacteria detected. In contrast, two control mice died in this 24-h period, and among those surviving, all had persistent and high PA levels in BAL  The experiments showed that phages administered in the three approaches all reduced BAL cytokine levels. The three approaches showed no PA growth in cultures obtained from phage-treated infected mice spleen. No evidence of murine toxicity following rapid phage-induced PAO1 and CF PA lysis | PA 12B-4973 was unusable in the delayed and prophylactic approaches owing to its virulence | Lack of negative control  No *in vitro* experiments were done to retest PA colonies recovered from mice receiving delayed or prophylactic phage doses, hence phage susceptibility and phage resistant PA strains remain unknown  Only one virulent CF strain tested in a single experimental condition owing to problems related to virulence | Evidence on the BAL benefits induced by phage treatment in PAO1 and CF PA-infected mice provides support for designing future human clinical trials in patients with CF  Evaluating inflammatory cell levels in mice BAL and mice spleen cultures, provides reliable evidence on PA infection and dissemination |
| --- | --- | --- | --- | --- | --- | --- | --- | --- | --- | --- |

*Abbreviations: CF, cystic fibrosis; NA, data not available; PBS, phosphate buffered saline; BAL, bronchoalveolar lavage; *in accordance with the International Committee on Taxonomy of Viruses. Available at:* [*https://talk.ictvonline.org/taxonomy/*](https://talk.ictvonline.org/taxonomy/)*; **results in vitro in* ***Additional file 1****; ^***^MOI, multiplicity of infection = the ratio between the plaque-forming units (PFU) and the colony-forming units (CFU);^****^EOP, efficiency of plating = the ratio between the average PFU on target bacteria and average PFU on host bacteria; ^†^poster presented at the European Congress of Clinical Microbiology and Infectious Diseases 2016. No published results.*
