# Supplementary material for: Challenges and Promises for Planning Future Clinical Research Into Bacteriophage Therapy Against Pseudomonas aeruginosa in Cystic Fibrosis. An Argumentative Review
Source: Front Microbiol. 2018 May 4;9:775. doi: 10.3389/fmicb.2018.00775 (PMC5945972; doi:10.3389/fmicb.2018.00775)
Supplement: Supplementary file 3 [file Table_1.docx]

| **Table 1 \| *Pseudomonas aeruginosa* (PA) phenotypes, infection stages, virulence factors, evolution, biofilm production and composition, metabolic impact, physiopathology, and survival strategies in cystic fibrosis (CF) lungs.** | | | | |
| --- | --- | --- | --- | --- |
|  | | | | |
| **PA phenotypes (CF infection stage)*** | **PA virulence factors, and evolution** | **PA biofilm** production, composition, and metabolic impact** | **Typical CF lung physiopathology, and PA survival strategies** | **References (first author and publication year)** |
| Environmental non-mucoid PA, wild-type (first acquisition) | Lipopolysaccharide capsule, adhesin, flagella, fimbriae, extracellular enzyme production (i.e. elastase, proteases, exotoxins) | PA biofilm is lacking, and planktonic cell PA are fluctuant (free-swimming) | Thick dehydrated mucus causes muco-ciliary dyskinesis, and progressively favors PA biofilm production | Anderson, 2012;  Chmiel and Davis, 2003;  Gellatly and Hancock, 2013 |
| Non-mucoid and mucoid PA (intermittent and recurrent infection) | Mucoid PA is able to overproduce alginate and biofilm matrix | Biofilms in tower- and mushroom-like micro-colonies containing embedded self-aggregated cells in a sessile form, and embedded in a matrix including DNA, polymers (i.e. F-actin) from dying neutrophils, extracellular polymeric substance (EPS) (containing three polysaccharides: alginate, Pel and Psl), proteins and extracellular DNA increasing biofilm volume, viscosity and progressive increase in growth  Biofilm preserves PA from leukocyte defensive action, reactive oxygen species, and nitric oxide  PA planktonic cells can leave the biofilm, enter the bulk fluid and colonize new lung sites thus initiating new sessile PA micro-colonies | Progressive muco-ciliary dyskinesis and biofilm production and overproduction | Battán et al., 2004;  Chmiel and Davis, 2003;  Cochran et al., 2000;  Costerton et al., 1999, 2003;  Davies et al., 1989;  Donlan and Costerton, 2002;  Furukawa et al., 2006;  Gibson et al., 2003;  Høiby et al., 2010;  Jesaitis et al., 2003;  Parks et al., 2009;  Rasamiravaka et al., 2015;  Walker et al., 2005;  Wei and Ma, 2013;  Yoon and Hassett, 2004 |
| Non-mucoid converted into mucoid PA, or small-colony variant PA (chronic colonization) | Small-colony variant PA are slow growing, hyperpiliated, hyperadherent, autoaggregative, and overproduce alginate  PA evolutionary adaptation and diversification by gene mutations involved in lipopolysaccharide and pyoverdine biosynthesis, exotoxin A regulation, flagella-lacking and transcriptional regulation (quorum-sensing) factors. All these changes enable PA to persist and survive and reduce its acute virulence (metabolic fitness) |  | Owing to lack of oxygen and nutrients, the CF lung environment affects PA metabolism, and influences oxygen-requiring drug activity and metabolically active cells  PA mutations, favor PA persistence in the lungs, immune evasion capacity, support the PA long-term survival “insurance hypothesis”, and PA antibiotic resistance in biofilm can become up to 1.000 fold higher than the PA planktonic counterpart | Boles et al., 2004;  Gooderham and Hancock, 2009;  Mah and O’Toole, 2001;  Moskowitz et al., 2004;  Smith et al., 2006;  Starkey et al., 2009;  Walters et al., 2003;  Winstanley et al., 2016;  Xu et al., 2000 |

*Note: *in the various cystic fibrosis infection stages, Pseudomonas aeruginosa evolution, biofilm lifestyle, metabolic impact and survival strategies could overlap; **biofilms exhibit a lifestyle that allows Pseudomonas aeruginosa (and other Gram-negative and Gram-positive bacteria) to grow and survive, aggregate cells in communities encased within a matrix adherent to the lung surface in cystic fibrosis, and resist immunological and antimicrobial attacks.*
