# Supplementary material for: Challenges and Promises for Planning Future Clinical Research Into Bacteriophage Therapy Against Pseudomonas aeruginosa in Cystic Fibrosis. An Argumentative Review
Source: Front Microbiol. 2018 May 4;9:775. doi: 10.3389/fmicb.2018.00775 (PMC5945972; doi:10.3389/fmicb.2018.00775)
Supplement: Supplementary file 4 [file Table_2.docx]

| **Table 2 \| Lytic bacteriophage (phage) classification, characteristics*, and phage families tested, and not tested *in vitro* and *in vivo* experiments on *Pseudomonas aeruginosa* (PA) strains isolated from patients with cystic fibrosis (CF).** | | | | |
| --- | --- | --- | --- | --- |
| **Order**** | **Family** (structure, %)** | **Nucleic acid (genomic arrangement)** | **Phage families tested (%) in *in vitro* and *in vivo* studies on PA strains** | **References** |
| *Caudovirales* (tailed phages) | *Myoviridae* (long, rigid and contractile tail, 25%); *Podoviridae* (short non-contractile tail, 14%); and *Siphoviridae* (long, flexible, no contractile tail, 61%)  Phages have icosahedral heads | Double-stranded (ds) DNA (linear) | *Myoviridae* (41%) and *Podoviridae* (38%) tested on laboratory PA (i.e. PAO 1), CF and non-CF PA strains; *Siphoviridae* (20%) tested on laboratory PA strains | Ackermann, 1998;  Ackermann, 2011;  Pires et al., 2015;  Tiwari et al., 2011 |
| Unassigned (tailless phages) | *Microviridae, Corticoviridae, Tectiviridae, Leviviridae, Cystoviridae* (enveloped virions)  Phages have icosahedral cubic symmetry or related bodies | dsDNA (linear, circular or supercoiled) or single-stranded (ss) DNA (circular); ssRNA (linear) or dsRNA (multipartite linear) | Unused in study *in vivo* on PA | Ackermann, 2011 |

**modified by Ackermann, H. W. (2005). “Bacteriophage classification” in Bacteriophages: Biology and Applications, eds. E. Kutter and A. Sulakvelidze (Boca Raton, FL: CRC Press), 169-187; **in accordance with the International Committee on Taxonomy of Viruses. Available at:* <https://talk.ictvonline.org/taxonomy/>
