# Supplementary material for: Challenges and Promises for Planning Future Clinical Research Into Bacteriophage Therapy Against Pseudomonas aeruginosa in Cystic Fibrosis. An Argumentative Review
Source: Front Microbiol. 2018 May 4;9:775. doi: 10.3389/fmicb.2018.00775 (PMC5945972; doi:10.3389/fmicb.2018.00775)
Supplement: Supplementary file 5 [file Table_3.docx]

| **Supplementary Table 3 \| Line of arguments from 20 studies included in the review investigating *in vitro* lytic bacteriophage (phage) effects against laboratory *Pseudomonas aeruginosa* (PA) strains or non-cystic fibrosis (CF) strains or PA strain hosts isolated from patients’ wounds, patients with diseases other than CF, and patients with CF (studies reported alphabetically according to the first authors’ surnames).** | | | | | | | | | | |
| --- | --- | --- | --- | --- | --- | --- | --- | --- | --- | --- |
| **First author, year (Country)** | **Phage taxonomy (family)**  **[phage bank and genome sequence accession numbers]*** | ***Pseudomonas aeruginosa* (PA) strain hosts** | **Methods used to assess phage host range against PA** | | **Key results from experiments after assessing lytic phage efficacy against PA** | **Problems reported on safety and efficacy** | **Limitations to overcome in future research on CF PA** | | **Reliable and repeatable findings useful for planning future CF clinical research *in vitro*** | |
| 1- Alemayehu, 2012 (Ireland)** | Two newly-isolated φNH-4 (*Myoviridae*) and φMR299-2 (*Podoviridae*)  [GenBank numbers: JN254800, φNH-4; JN254801, MR299-2] | *Lux-*tagged PA strains NH57388A (mucoid) and MR299 (non-mucoid) isolated from patients with CF | Plaque assay to determine host range against 10 CF PA strains from University College Cork, Cork University Hospital, Alimentary Health Ltd. (Cork, Ireland) | | A mix of two phages reduced bacterial cell numbers in PA biofilms grown on a bronchial epithelial cell monolayer from patients with cystic fibrosis. The phage-mix decreased the risk of resistant mucoid and non-mucoid PA colonies | None | Only two phages tested | | Studies *in vitro* need to test phage cocktail therapy in mucoid and non-mucoid *lux-*tagged PA biofilms grown on a bronchial epithelial cell monolayer from patients with CF | |
| 2- Alves, 2015 (United Kingdom) | Six newly-isolated phages: DL52, DL60, DL68 (Myoviridae), DL54, DL62 and DL64 (*Podoviridae*)  [GenBank numbers: KR054028, DL52; KR054030, DL60; KR054033, DL68; KR054029, DL54; KR054031, DL62; KR054032, DL64] | PAO1 strain isolated from a patient’s wound | Spot test | | A phage cocktail was more effective than a single phage in decreasing planktonic PAO1 culture and biomass in biofilm models  In the planktonic PAO1 culture, the phage cocktail exhibited a more positive and rapid effect in eliminating the PA load in broth cultures than in PA biofilms  Under static conditions, the bacterial load was disrupted at a lower rate than the load in the planktonic PAO1 culture, and biofilm cells regrew  Biofilms took longer to disrupt under dynamic flow conditions than under static conditions, and no PA regrowth appeared within the timeframe of the experiment | None | The phage cocktail was tested only on laboratory PAO1 strain | | To mimic a more realistic infection situation active phage replication on PAO1 biofilm should be checked under dynamic flow conditions | |
| 3- Betts, 2013 (France) | PEV2, LUZ7, LKD16 (*Podoviridae*), 14/1 (*Myoviridae*)  [GenBank numbers: KU948710.1, PEV2; FN422398, LUZ7; AM265638.1, LKD16; data for phage 14/1 unreported and irretrievable] | PAO1 strain isolated from a patient’s wound | No host range reported | | Phage activity improved through a standard training process (at least 5-6 phage passages on the host tested)  During phage training, bacteria became resistant to ancestor phages and cross-resistance developed to other untested (foreign) phages | Resistant PA strains developed | PA resistance was evaluated only after the first of the serial passages used for the standard training procedure | | PA bacterial resistance and cross-resistance to other untested phages should be evaluated when phage standard training ends | |
| 4- Coulter, 2014 (Texas, USA) | PB-1 (*Myoviridae)*  [The study reported ATCC^®^ number 15692-B3^™^; GenBank number EU716414.1] | PAO1 strain isolated from a patient’s wound | Host range evaluated with an unspecified test | | Tobramycin and PB-1 given simultaneously was as effective as tobramycin alone in decreasing PA biofilm mass  Phage and antibiotic treatment combined reduced PA resistance | None | The bactericidal antibiotic tobramycin, given simultaneously to the phage, inhibited PA protein synthesis, thus interfering with phage production | | Potential phage-antibiotic interactions should be tested after assessing useful predefined tobramycin and phage concentrations. In future studies tobramycin and phage therapy treatments should be given sequentially to avoid the tobramycin bactericidal effect induced by simultaneous administration with phages | |
| 5- Danis-Wlodarczyk, 2015 (Poland) | Two newly-isolated KT28 and KTN6 (*Myoviridae*)  [GenBank numbers: KP340287, KT28; KP340288, KTN6] | PAO1 and PA0038 strains isolated from a patient’s wound, and PA708 isolated from a patient with CF | Host range evaluated with an unspecified test | | The various tests used showed that the phage cocktail effectively stimulated PA biofilm eradication activities. Crystal violet (CV) staining and colony-forming unit (CFU) counts detected a 70-90 bacterial cell reduction in 24-72 h-old biofilm; spectrophotometry (SPM) and fluorometry (FM) detected a reduction in pyocyanin and pyoverdine secretions, derived from PA biofilm degradation; laser interferometry (IFM) and goniometry (GM) after phage application detected in PA01 strain an increase in biofilm structure disruption products  No phage mutants appeared in PA-resistant host variants | After active KT28 and KTN6 phage treatment for one day, more than 90% of persistent PA cells became insensitive to both phages, and cross-resistance developed | No information on whether PA 708, isolated from a patient with CF, was a mucoid or non-mucoid phenotype or the patient with CF had a chronic PA infection | | A composite standard reference test (including CV, CFU, SPM, FM, IFM and GM) should be used to detect phage cocktail efficacy on PA biofilm eradication  Phage cocktail treatment on PA hosts must be prolonged over 24 h to detect possible PA cells insensitive to phages, and cross-resistance | |
| 6- Danis-Wlodarczyk, 2016 (Poland)** | One newly-isolated KTN4 (*Myoviridae*)  [GenBank number: KU521356] | PAO1 and non-CF 0038 PA strains isolated from a patient’s wound, and the small colony variant PA strain CF708 isolated from a patient with CF | Host range evaluated with an unspecified test against 58 clinical strains from Military Hospital Neder-Over-Heembeek, Brussels, Belgium | | The KTN4 phage had a strong bactericidal effect against PA strains, as tested in a gentamicin exclusion assay on airway surface liquid (ASL) models  CV and several well-controlled, standard reference tests showed that pigmented signaling molecules secreted by the PA hosts decreased significantly, proving that the KNT4 phage was strongly potent in PA biofilm treatment  The KTN4 phage freely diffused and gained access to the PA hosts in ASL models, but treatment success depended strongly on the PA strain features  Colistin and phage, given simultaneously, had no synergistic effects | In a CV binding assay phage treatment increased the biofilm biomass | Colistin caused PA cell deaths by destabilizing cell membrane, thus limiting phage propagation | | CV and several well-controlled standard reference tests, and standardized PA host conditions, mimicking normal and CF lung environments, should test phage effect treatment combined with antibiotics on PA biofilms  To design experimental *in vivo* studies, KTN4 phage bactericidal effect combined with antibiotics, and PA host-pathogen interactions should be tested *in vitro* by using the gentamicin exclusion assay on an ASL *in vitro* model that is flexible, and generates reproducible data on non-CF and CF epithelial cell lines  To avoid a colistin bactericidal effect, *in vitro* experiments combining phage and colistin, the antibiotic should be given sequentially and not simultaneously | |
| 7- Essoh, 2013 (France) | 6 Pyophage-derived phages: P1-14_pyo_ (Myoviridae), P1-15_pyo_, P8-13_pyo_,, P2-10_pyo_, P3-20_pyo_, PTr60_pyo,_ (*Podoviridae*)  [data unreported and irretrievable]  Newly-isolated phages: P1-14_Or01_ (Podoviridae) and P2-10_Ab01_ (*Myoviridae*)  [EMBL-EBI numbers: HE983844, P1-14_Or01;_ HE983845, P2-10_Ab01_] | Forty-seven PA strains from different clusters isolated from patients with CF | Spot test | | Efficient cocktails contained several phage genera, but a phage cocktail that efficiently reduced all PA strains was difficult to prepare  Phages behaved similarly alone, or in a cocktail  None of the 13 phage-resistant PA strains contained the clustered, regularly interspaced, short palindromic repeat (CRISPR)-Cas system | The combined pyophage preparation or each newly-isolated phage individually failed to lyse 13 of the 47 PA strains | Bacteria sensitivity and phage host range determined by phage spotting on a lawn of stationary growing bacteria. This metabolic bacterial status could reduce phage lytic activity as others have shown (Abedon and Yin, 2009; Middelboe, 2000; Sillankorva et al., 2004) | | Experiments to test phage cocktail efficiency, should evaluate the presence of the PA resistance mechanism CRISPR-Cas in several PA-resistant strains  Care is needed to avoid the metabolic PA status owing to a lawn of stationary growing bacteria that might reduce phage lytic activity | |
| 8- Friman, 2016, (United Kingdom) | 14/1, phiKZ, PT7 (*Myoviridae*) and PNM (*Podoviridae*)  [GenBank number: NC_004629.1, phiKZ; data unreported and irretrievable for phages 14/1, PT7 and PNM] | Ten PA strains isolated from patients with CF (five with intermittent, and five with chronic infections) | Host range not evaluated | | Trained (evolved) phages were more efficient than ancestral phages in reducing PA bacterial densities, whereas this effect was greater and more consistent when bacteria strains originated from chronic than from intermittent infections  Phage selection, during *in vitro* phage bacterial coevolution, led to decreased bacterial growth (fitness cost) measured in the absence of phages only for chronic isolates  PA became resistant to ancestral and evolved phages at the end of the *in vitro* phage-PA co-evolution procedure (5-days) | No difference was found among mean PA-resistance levels tested *in vitro* with the various trained phages  The phage effect differed considerably between different PA isolates. This variation was more evident within intermittent than in chronic PA isolates | Limited number of PA strains used despite the considerable existing strain variation within and between individual patients with CF | | Trained (evolved) phages should be selected by estimating the decrease in phage-resistant PA growth (fitness cost), especially comparing intermittent and chronic PA infections  Experiments should evaluate PA resistance to ancestral and trained phages when phage phage-PA co-evolution ends | |
| 9- Garbe, 2010 (Germany) | Newly-isolated JG024 (*Myoviridae*)  [GenBank number: GU815091] | PAO1, PA mucA mutant strain (mucoid variant of PAO1) and mucoid BT73 strain (isolated from a patient with CF) | Spot test to determine host range against 19 clinical isolates from CF patients, and from urinary tract infections as well as a collection of 100 environmental strains | The BT73 CF mucoid strain infected by phage JG024 *in vitro* showed lower susceptibility to phage infection than PAO1 and PA mucA mutant strains  An artificial sputum medium (ASM) mimicking CF lung infection conditions was a suitable method for investigating whether phages lysed PA strains  In accordance with the hypothesis that alginate produced by mucoid strains can reduce phage activity, adding alginate to Luria Bertani (LB) broth reduced phage infection efficiency | | None | | Phage activity in ASM was tested against a single CF strain  No information on whether the BT73 CF mucoid strain had been isolated from a CF patient with a chronic PA infection | | In experiments testing phages and mucoid PA hosts in LB broth, alginate should be added, to provide evidence that alginate overproduction can influence phage efficiency in intermittent and in chronic PA infections |

| 10- Hall, 2012 (United Kingdom)*** | 14/1, phiKZ, PT7 (*Myoviridae*) and PNM (*Podoviridae*)  [GenBank number: NC_004629.1, phiKZ; data unreported and irretrievable for phages 14/1, PT7, and PNM] | PAO1 strain isolated from a patient’s wound | Host range not evaluated | Multi-phage therapy (simultaneous application) was more successful than single-phage therapy (sequential application) for reducing PA bacterial densities (estimated by optical density measurements), and caused no significant increase in the frequency of multi-resistance. Phage-resistant PA emerged in all experimental treatments, and incurred significant fitness costs, expressed as reduced growth rate in the absence of phages | Phage-resistant PA emerged | PA bacterial densities estimated by optical density measurements were probably altered by phage-resistant PA genotypes overproducing alginate or extracellular polymeric substances | To estimate simultaneous application of multi-phage therapy efficiency, phages should be selected by estimating the decrease in phage-resistant PA growth (fitness cost in CF PA clinical strains |
| --- | --- | --- | --- | --- | --- | --- | --- |
| 11- Hanlon, 2001 (United Kingdom) | Lysogenic phage F116 (*Podoviridae*) and GL1 (NA)  [GenBank number: AY625898.1, F116; data unreported and irretrievable for phage GL1] | The non-CF PA NCIMB 10548 and a PA mucoid strain from a patient with CF | Host range not evaluated | During incubation, even at the highest alginate concentration used (12% weight/volume), phage diffused through alginate gel. At the lower concentrations of commercial alginate and in the presence of purified CF PA exopolysaccharide, the phage rapidly diffused.  Despite the presence of exopolysaccharide, the phage reduced the number of viable bacteria in a biofilm by up to 99%  Younger biofilms seemed no more sensitive to phages than 20-day-old PA biofilms. PA biofilms of all ages yielded the same log-reduction factors | Cells grown planktonically in liquid cultures exhibited log-reduction factors greater than 5, suggesting that the re-suspended biofilm-derived cells still had increased resistance to treatment | Phage activity against old biofilm tested using only a laboratory PA strain instead of the CF PA strain | Future clinical research should avoid using lysogenic phages (*i.e*. F116)  Experiments should test phage ability to diffuse throughout alginate derived from mucoid PA strain hosts from patients with CF |

| 12- Henry, 2013 (France)** | Nine newly-isolated phages: PAK_P1, PAK_P2, PAK_P3, PAK_P4, PAK_P5 (*Myoviridae*) hosted on the PAK strain; PhiKZ (*Myoviridae*), and LUZ19 (*Podoviridae*) amplified on PAO1; CHA_P1 hosted on the PA CHA strain; LBL3 (*Myoviridae*) hosted on the Aa245 strain  [GenBank numbers: KC862297, PAK_P1; KC862298, PAK_P2; KC862299, PAK_P3; KC862300, PAK_P4; KC862301, PAK_P5; NC_004629.1, PhiKZ; NC_010326.1, LUZ19; KC862295, CHA_P1; NC_011165.1, LBL3] | The laboratory PAK, PAK-lumi (PAK bioluminescent version) and PAO1 strains, the multidrug-resistant-mucoid PA CHA strain isolated from a patient with CF, and the Aa245 strain isolated from a burn wound | Host range not evaluated | Efficiency of plating (EOP)^****^used to evaluate phage lytic efficacy on PAK lumi strain *in vitro* predicted the *in vivo* efficacy of therapeutic phages against PA infections, and *in vitro* and *in vivo* EOP results showed a good correlation | None | The experiments testing phage activity by EOP were conducted using the laboratory PAK-lumi strain and not a CF strain host | Experiments designed to compare the lytic activity of each phage with the activity observed on PA hosts should use the EOP test *in vitro* rather than simple spot tests |
| --- | --- | --- | --- | --- | --- | --- | --- |
| 13- Larché, 2012 (France) | Three newly-isolated phages: A, B, C (NA)  [data unreported and irretrievable] | Forty-four multi-drug-resistant (MDR) and extensively-drug-resistant (XDR) PA strains belonging to different clonal complexes from patients with unspecified pathologies | Spot test to determine host range against 30 PA isolates from Narbonne hospital and 8 from Percy hospital (France), and 6 from Korea | Forty-two MDR and XDR PA strains (95.4%) displayed high susceptibility to at least one phage, as well as to the phage cocktail | Two isolates belonging to the same lineage (representing alone 50% of all MDR isolates), showed resistance to all three phages, both individually and in cocktail | No complete information on DNA analysis to define whether the phages tested were lytic  MDR and XDR PA strains of unspecified origin  PA strain resistance evaluated from different laboratories from different countries, thus reducing generalizability of the results | Phage cocktail efficacy should be tested with MDR and XDR PA strain hosts from patients with CF |
| 14- Lehman, 2016 (United Kingdom) **^†^ | Four newly-isolated phages combined in the cocktail AB-PA01 (NA)  [data unreported and irretrievable] | Three-hundred sixty-nine PA strains from patients with CF, and 60 PA strains from non-CF patients, collected from 2007 to 2015. PA isolates included both antibiotic susceptible/resistant and mucoid/non-mucoid strains | Spot test to determine host range against 67 distinct CF strains | The phage-mix infected both antibiotic susceptible or resistant, and mucoid or non-mucoid CF PA strain hosts, showing that AB-PA01 had a broad range of activity on PA strains | A total 12.2% CF PA strains were insensitive to the phage cocktail | PA susceptibility to phages tested only by spotting phages on a lawn of PA in an unspecified metabolic status | Because the cocktail AB-PA01 has a broad range of activity on PA strain hosts it should be tested under dynamic metabolic conditions on mucoid or non-mucoid CF PA strains susceptible or resistant to antibiotics |

| 15- Lim, 2016 (Republic of Singapore) | PB1 (*Myoviridae*)  [GenBank number: EU716414.1] | | PAO1 strain isolated from a patient’s wound | Host range not evaluated | The small colony variant (SCV) isolated from the surviving population of wild-type PAO1 strain exposed for 24 h to PB1 phage expressed little pyocyanin and elastase indicating that the *las* quorum sensing system was deficient in this strain | | None | Only one laboratory PAO1 strain tested | Because SCV PA strains express less pyocyanin and elastase than CF PA strains, experiments to detect phage resistance mechanisms in SCV PA hosts should include SCV phenotypic assays, DNA microarrays and whole-genome sequencing to highlight possible useful findings for patients with CF |
| --- | --- | --- | --- | --- | --- | --- | --- | --- | --- |
| 16- Olszak, 2015 (Poland)** | 28 newly-isolated phages, 2 of which were characterized: PA5oct and KT28 (*Myoviridae*)  [data unreported and irretrievable] | | A total 121 PA strains (including 29 mucoid strains differing in virulence) isolated from patients with CF, PAO1 and a clinical non-CF strain | Spot test to determine host range against all 121 CF strains tested | PA host features influencing phage activity were slow growth rate, low biofilm production, reduced twitching motility and chemical composition | None | | Unspecified PA infection stage | To determine whether PA host features (including slow growth rate, low biofilm production, reduced twitching motility and chemical composition) influence phage activity, these features should be determined in CF and non-CF PA strain hosts isolated from PA colonized patients |
| 17- Pires, 2011 (Portugal) | | Newly-isolated: phiIBB-PAA2, phiIBB-PAC23, phiIBB-PACL12, phiIBB-PAP21 (*Podoviridae*)  [data unreported and irretrievable] | PAO1 strain isolated from a patient’s wound, and an ATCC 10145 strain (unspecified origin) | Spot test to determine host range against 35 strains of PA | When treated with phages, PA biofilm initially reduced biomass and then regrew  Phages were equally efficient towards stationary and exponential phase PA cells | Phages isolated from clinical PA strains had a reduced host range  Some broad lytic phages failed to infect planktonic cultures  PA planktonic cultures and biofilm could become phage-resistant after the first 6-10 hours  Higher amounts of the phiIBB-PAA2 than the phiIBB-PaP21 phage became entrapped in the PA biofilms after 2-hour infection | | The origin of the ATCC 10145 strain remained unspecified | To evaluate phage resistance, PA planktonic cultures and biofilm should be tested after incubating phage cocktails for 6-10 hours  Avoid using the phiIBB-PAA2 rapidly entrapped in a PA biofilm  To demonstrate their presence and active replication during treatments, phage levels in PA biofilm should be checked 2-hour**s** after infection |

| 18- Saussereau, 2014 (France) | | PAK_P1, PAK_P2, PAK_P3, PAK_P4, PAK_P5, P3_CHA, CHA_P1, phiKZ, LBL3 (*Myoviridae*) and LUZ19 (*Podoviridae*)  [GenBank numbers: KC862297, PAK_P1; KC862298, PAK_P2; KC862299, PAK_P3; KC862300, PAK_P4; KC862301, PAK_P5; HM173081, P3-CHA; KC862295, CHA_P1; NC_004629.1, phiKZ; NC_011165.1, LBL3; NC_010326.1, LUZ19] | Twenty small or large PA-colonies, including mucoid and non-mucoid PA strains, from four aliquots collected from 48 sputum samples from patients with CF in three French hospitals | Host range not evaluated | None of the clinical parameters, retrospectively checked in patients with CF, were associated, either positively or negatively, with phage efficacy on their sputum samples infected with PA strains. Hence the microenvironment in the lungs of patients with CF had no influence on phage efficacy (“patient-independent efficacy”)  The most efficient phages *in vivo* were those isolated ‘deliberately’, using patients' PA strains, supporting the use of a personalized approach to achieve optimal treatment | None | Inadequate information on phage lytic activity evaluated on mucoid and non-mucoid PA collected from CF patients’ sputum in a selective medium | To test phage efficacy the correlation with clinical criteria should be avoided  The most efficient technique to yield evidence on formulating new phage cocktails efficient for CF is that deliberately isolating phages on PA patients’ strains (personalized approach) |
| --- | --- | --- | --- | --- | --- | --- | --- | --- |
| 19- Torres-Barceló, 2014 (France) | LUZ7 (*Podoviridae*)  [GenBank number: FN422398] | | PAO1 isolated from a patient’s wound | Host range not evaluated | Phage at a multiplicity of infection (MOI)^§^ unspecified and streptomycin treatments (at the dose100 or 240 µg/mL) were tested on PAO1 at different time points: simultaneously, or streptomycin at 12 and 24 h after phage administration  Phage and streptomycin treatment combined acted synergistically, given that after adding the two treatments singly, combined treatment lowered bacterial density more than expected  The key for minimizing PA resistance levels depended not on the antibiotic dose but on applying antibiotics at the peak phage efficacy, and not simultaneously  Combined treatment provided no evidence for a trade-off between antibiotic resistance and phage resistance. Higher phage resistant levels were associated with higher antibiotic resistance | None | MOI unspecified | In *in vitro* studies studying independent and synergistic effect of combined phages and antibiotics should predefine sub-lethal and MIC antibiotic concentrations for PA hosts  To obtain the optimal treatment window, antibiotics should be given 12 h after phages  To detect phage-resistance PA hosts bacteria should be exposed to prolonged treatments for 70 h  Prediction on phage application should be determined on a MOI-based finding |

| 20- Uchiyama, 2016 (Japan) | Newly-isolated KPP22 (*Myoviridae*)  [GenBank number: LC105987] | PAO1 isolated from a patient’s wound | Host range not evaluated | EOPs of ancestral KPP22 phage on the KPP22-PAO1 resistant clones were significantly lower (ca. 10^−4^ to 10^−5^ times) than that on the laboratory PAO1 host strain. In contrast, the EOPs of the three mutant KPP22 phages on the KPP22-resistant PAO1 clones showed the same efficacy expressed against the laboratory PAO1 host strain | None | The encouraging results obtained by testing mutant phages need to be replicated in CF PA from chronic pulmonary infections | Phage infectivity levels should be examined by comparing EOP in ancestral and mutant phages  The best mutant phages efficient against phage-resistant PA hosts should be detected and reported by genetic analysis and EOP |
| --- | --- | --- | --- | --- | --- | --- | --- |

*Abbreviations: CF, cystic fibrosis; NA, data not available; *in accordance with the International Committee on Taxonomy of Viruses. Available at:* [*https://talk.ictvonline.org/taxonomy/*](https://talk.ictvonline.org/taxonomy/)*; phage bank and genome sequence accession* *numbers for 8 papers (Betts, 2013; Coulter, 2014; Friman, 2016; Hall, 2012; Hanlon, 2001; Lim, 2016; Saussereau, 2014; Torres-Barceló, 2014) retrieved by the review authors; **results in vivo reported in* ***Supplementary Table 4****; ^§^MOI, multiplicity of infection = the ratio between the plaque-forming units (PFU) and the colony-forming units (CFU); ^***^ the in vivo results from this study were excluded because they exclusively referred to PAO1 infecting a non-pulmonary animal model; ^****^EOP, efficiency of plating = the ratio between the average PFU on target bacteria and average PFU on host bacteria; ^†^poster presented at the European Congress of Clinical Microbiology and Infectious Diseases 2016. No published results.*
