# Supplementary material for: Challenges and Promises for Planning Future Clinical Research Into Bacteriophage Therapy Against Pseudomonas aeruginosa in Cystic Fibrosis. An Argumentative Review
Source: Front Microbiol. 2018 May 4;9:775. doi: 10.3389/fmicb.2018.00775 (PMC5945972; doi:10.3389/fmicb.2018.00775)
Supplement: Supplementary file 6 [file Table_4.docx]

| **Supplementary Table 4 \| Line of arguments from 9 studies included in the review investigating *in vivo* lytic bacteriophage (phage) effects against laboratory *Pseudomonas aeruginosa* (PA) strains or non- cystic fibrosis (CF) strains or PA strain hosts isolated from patients’ wounds, patients with diseases other than CF, and patients with CF in pulmonary and non-pulmonary host models (PA-infected animal models) (studies reported alphabetically according to the first authors’ surnames).** | | | | | | | | | | |
| --- | --- | --- | --- | --- | --- | --- | --- | --- | --- | --- |
| **First author, year (Country)** | **Phage taxonomy (family)**  **[Phage bank and genome sequence accession numbers]*** | **PA strain(s) used in the experiments** | **Animal host models tested** | **Phage host range methods used to assess lytic activity against PA** | **Products used for suspending phages** | **Control group treatments** | **Key results** | **Problems reported in testing safety and efficacy** | **Limitations to overcome in future research on CF** | **Reliable and repeatable findings useful for planning future CF clinical research *in vivo*** |
| 1- Alemayehu, 2012 (Ireland)** | Two newly-isolated φNH-4 (*Myoviridae*) and φMR299-2 (*Podoviridae*)  [GenBank numbers: JN254800, φNH-4; JN254801, MR299-2] | *Lux*-tagged PA strains NH57388A (mucoid) and MR299 (non-mucoid) isolated from patients with CF | Mice | Phage plaque assay on 10 CF strains from University College Cork, Cork University Hospital, Alimentary Health Ltd. (Cork, Ireland) | PBS solution | Infected control group received phosphate-buffered saline (PBS) instead of phage mix | The two-phage mix is effective in killing *lux*-tagged PA in the lungs of infected mice | None reported | Lack of a negative control group. The study tested only a two-phage cocktail | Studies in animal models need to test phage cocktail efficiency on mucoid and non-mucoid *lux*-tagged CF PA strains |
| 2- Beeton, 2015 (United Kingdom) | Newly-isolated DL52, DL60, DL68 (*Myoviridae*). DL54, DL62, DL64 (*Podoviridae*)  [GenBank numbers: KR054028, DL52; KR054030, DL60; KR054033, DL68; KR054029, DL54; KR054031, DL62; KR054032, DL64] | PAO1, PA45291 (isolated from bacteremia) and BC09007 (isolated from a patient with CF) | *Galleria mellonella* larvae | No tests used | Not reported | Three control groups, two negative (one of PA uninfected- phosphate-buffered saline-PBS injected *Galleria mellonella* larvae, to assess the impact of any negative effect from the injection procedure; and one of PA uninfected phage-treated larvae to assess the toxicity of the phage cocktail), and one positive (PA infected larvae treated with PBS solution) | A 6-phage cocktail used in the preventive and curative approaches, prolonged survival of infected larvae in a dose-dependent manner  This study validates the use of *Galleria mellonella* as a simple, robust and cost-effective model for initial *in vivo* examination of PA-targeted phage therapy  The investigators suggest that continual PAO1 survival in the presence of a high phage titer could be caused by intracellular localization of PA, having ruled out phage resistance developing within the larvae  The efficacy of phage treatment is multiplicity of infection (MOI)**** related (the higher the MOI the better the result)  Susceptibility reassessed in re-isolated PAO1 | Phage-treated *Galleria mellonella* infected by PAO1 died at 30 h post-infection and larvae infected by clinical strains PA45291 and BC09007 eventually succumbed at 40 h | The authors failed to provide information on why they used phage suspensions at MOI 100 only for the preventive model, but presumably owing to experimental risks including killing the larvae | Reassessing susceptibility in re-isolated PAO1 will rule out possible PAO1 phage resistance during *in vivo* infection |

| 3- Danis-Wlodarczyk, 2016 (Poland)** | Newly-isolated KTN4 (*Myoviridae*)  [GenBank number: KU521356] | PAO1, non-CF0038 strains isolated from a patient’s wound, and the small colony variant CF708 strain isolated from a patient with CF | *Galleria mellonella* larvae | Unspecified phage efficacy test on 58 clinical PA strains (from the Military Hospital Neder-Over-Heembeek, Brussels, Belgium) | Not reported | The controls consisted of PA uninfected *G. mellonella* larvae receiving phage lysate only (negative), and larvae infected with bacterial lethal dose (positive) | The KTN4 phage has an antibacterial strain-dependent efficacy against clinical isolates  Infected larvae were incubated for up to 120 hours | None reported | Because it grows slowly and leads to slow biofilm formation, low type IV pili expression, and lower virulence than PAO1 and non-CF0038, the small colony variant CF 708 strain is not a representative PA strain colonizing patients with CF | Studies on slowly growing PA strains should incubate larvae for at least 120 hours |
| --- | --- | --- | --- | --- | --- | --- | --- | --- | --- | --- |
| 4- Debarbieux, 2010 (France) | Newly-isolated PAK-P1 (*Myoviridae*)  [GenBank number: KC862297] | Bioluminescent laboratory PAK (PAK lumi) strain | Mice | Efficiency of plating (EOP)^***^ test for PAK P1 phage on 10 CF PA strains from primary, and 10 CF PA from chronic colonization (French CF strain collection center) | PBS solution | For curative treatment: PA uninfected phage-treated mice (negative), PA infected-phosphate-buffered saline-treated (PBS)-mice (positive). For preventive treatment, PBS-pre-treated PA infected mice (positive) | Phage treatment is effective in saving mice from lethal infection in the curative model, and prevents lung infection when given 24 h before bacterial infection  Giving a bioluminescent PA intra-nasally quantifies efficacy by phage multiplication in the respiratory tract. Phages tend to persist in the lungs  Active phage efficacy in PA infected mice is dose and time-dependent, and a phage-to-bacterium ratio of 10:1 is harmless to mice. A solution of heat-killed PAK-P1 phage, given 2 h after PA infection, killed similar mouse numbers in the negative and positive control groups  To monitor phage treatment harmless to mice treated with MOI 100, 10 times higher than a standard dose, mice behaviour, fur and weight were monitored for 10 days.  To evaluate mouse lung infection status in PA phage-treated infected mice and in mouse controls lactate dehydrogenase enzyme levels were measured in BALs 6 h after the infection | None reported | The PAK P1 environmental phage effectively lysed in plates only 10% of PA from patients with chronic infection, and was effective in lysing 50% of clinical strains isolated from patients with primary colonization  Owing to high phage variability, completely annotating a phage genome requires in-depth bioinformatic analysis  In uninfected PA mice, phage replication showed a 2-log decrease in 24 h | Phage host range needs to be evaluated by EOP  Studies on preventing PA infection should use non-invasive techniques (i.e. bioluminescence)  To evaluate the safety of a high phage treatment dose, mice should be monitored for behavior, fur and weight for 10 days  In PA-infected mice, the maximum possible delay in phage treatment, for maintaining ~~a~~ 100% survival, is 2 hours  Studies assessing phage safety in infected PA mice should ensure a phage-to-bacterium ratio that is harmless to mice  Measuring lactate dehydrogenase enzyme levels (released from lung damaged cells) in mouse BALs at 6 h after PA infection will identify the time in which bacteria multiply fastest and bacterial phage susceptibility is highest |
| 5- Henry, 2013 (France)** | Nine newly-isolated phages: PAK_P1, PAK_P2, PAK_P3, PAK_P4, PAK_P5 (*Myoviridae*) hosted on the PAK strain; PhiKZ (*Myoviridae*), and LUZ19 (*Podoviridae*) amplified on PAO1; CHA_P1 hosted on the PA CHA strain; LBL3 (*Myoviridae*) hosted on the Aa245 strain  [GenBank numbers: KC862297, PAK_P1; KC862298, PAK_P2; KC862299, PAK_P3; KC862300, PAK_P4; KC862301, PAK_P5; NC_004629.1, PhiKZ; NC_010326.1, LUZ19; KC862295, CHA_P1; NC_011165.1, LBL3] | A bioluminescent laboratory PAK strain (PAK-lumi) | Mice | EOP test for 9 phages on PAK lumi strain | Not reported | Infected mice treated with PAK_P1 (MOI 0.1) or phosphate-buffered saline (PBS) | The good correlation between *in vitro* results and *in vivo* efficacy for 7 phages, gives a reliable index to predict phage efficacy. *In vitro* efficacy is insufficient to ensure *in vivo* efficacy. The optimal efficacy achieved with the 5 phages isolated on the PA strain used in the mouse model highlights a possible effect of the bacterial host used for isolation on the efficacy of the treatment.  Bioluminescence in mice was recorded by a real-time imaging system (specialized living image software) to monitor infection in  the whole lung | CHA_P1 phage (amplified on a clinical PA strain CHA) was unable to cure animals infected with the PAK-lumi strain, despite being genetically closely related to PAK_P3 and PAK_P5. In addition, an attempt to adapt this phage to the PAK strain failed | The experiments testing phage activity by EOP conducted using the laboratory PAK-lumi strain and not a CF strain as the phage host  Using phages isolated on patients’ PA strains is time consuming | Phage host range needs to be evaluated by EOP.  To validate results from studies *in vitro*, studies *in vivo* need to test cocktail phage therapy isolated on the same PA strains (i.e. personalized) used in mouse model treatment, and measure efficacy with real-time imaging system software |

| 6- Lehman, 2016 (United Kingdom)**^†^ | Four newly-isolated phages combined in the cocktail AB-PA01 (NA)  [data ureported and irretrievable] | Three hundred and 69 PA strains from patients with CF and 60 PA strains from non-CF patients collected between 2007 and 2015. Isolates included both antibiotic susceptible or resistant and mucoid or non-mucoid strains | Mice | Unspecified phage efficacy test on 67 PA strains from CF patients (unspecified collection center) | Not reported | Unspecified non-CF or CF PA-infected mice treated with the phage diluent | The study confirmed the usability of AB-PA01 for clinical use (exclusively lytic, efficacious *in vivo*), nebulization (no significant decreases in titers were observed) and long-term phage stability (GMP manufacturing current process optimization) | None reported in the poster | The PA strains used to infect mice were not specified. No negative control group reported  Failed to specify where the PA strains used for testing phage efficacy were collected | Studies on antibiotic susceptible or resistant and mucoid or non-mucoid PA strains need to test phage efficacy on several PA strains taken from patients with CF |
| --- | --- | --- | --- | --- | --- | --- | --- | --- | --- | --- |

| 7- Morello, 2011 (France) | P3-CHA derived and trained (evolved) from PAK-P3 (*Myoviridae*)  [GenBank: HM173081] | Multidrug-resistant (MDR) and mucoid PA CHA strain isolated from a patient with CF | Mice | EOP test for PAK-P3 and P3-CHA on 10 CF PA strains from primary, and 10 CF PA from chronic colonization (French CF strain collection center) | PBS solution | Curative treatment involved PA CHA infected- phosphate-buffered saline (PBS) treated mice (positive control) and PA CHA uninfected heat-killed P3-CHA treated mice (negative control).  Preventive phage treatment involved heat-killed P3-CHA pre-treated PA CHA infected mice (positive control) and P3-CHA pre-treated uninfected mice (negative control) | Curative phage treatment acts cooperatively with the mice immune response to eliminate acute lung infection caused by MDR PA CHA. Preventive phage treatment requires non-heat-killed active phages  Training (evolved) *in vitro* a phage towards a CF multidrug resistant clinical strain improves its efficacy in curative and preventive experiments *in vivo* | None reported | For curative treatment, phage concentration data reported in the table and in the results showed major discrepancies, thus determining possible reporting bias  The reported MDR PA strain used in the experiment, failed to adhere to the international MDR bacteria definitions (Sievert et al., 2013) | Phage host range needs to be evaluated by EOP  Studies testing i*n vivo* trained phage therapy on CF MDR PA strains need to adhere to international MDR bacteria definitions  Training (evolved) phage (using an endotoxin-free phage solution) *in vitro*, could avoid stimulating a host immune response that could mask the effects of phage curative and preventive treatments *in vivo* |
| --- | --- | --- | --- | --- | --- | --- | --- | --- | --- | --- |

| 8- Olszak, 2015 (Poland)** | 28 newly-isolated phages (only two characterized): PA5oct and KT28 (*Myoviridae*)  [data ureported and irretrievable] | PAO1, non-CF0038 strain and 4 clinical CF strains (CF217, CF708, CF532, CF832) with diverse degrees of virulence | *Galleria mellonella* larvae | Phage spot test on 123 PA isolates (PAO1, 121 clinical CF PA from Prague CF Center collection, non-CF0038 clinical PA strain from the Institute of Genetics and Microbiology collection, University of Wroclaw, Poland) | Not reported | Uninfected *Galleria mellonella* larvae, sham-infected larvae, larvae receiving phage lysate only (negative), PA infected-untreated larvae, and infected larvae treated with ultra-violet (UV)-inactivated phages (positive) | The efficacy of single phages or cocktails is strain related  Larval survival reflects phage lytic activity rather than host immune stimulation | Phage failure t**o** rescue larvae infected with the 3 weakly virulent CF isolates given at a high load able to cause larva infections could depend on the toxic compounds released during massive bacterial cell lysis after phage propagation, thus causing greater mortality in treated than in untreated larvae | Phages chosen for cocktail preparations were not selected for different host receptor affinity, hence they competed for bacterial receptors  Because the mixture was less effective in lysing PAO1 and non-CF0038 than single lytic phage injection, phages need to be selected rigorously  Because weakly virulent PA strains given at a high load release toxic compounds by phage lysis, PA strains need to be carefully selected to avoid severe systemic inflammatory response and death in the animal hosts | Phage cocktail formulations need to be chosen on the basis of phages not competing for bacterial receptors  Active phages rather than UV-inactivated phages need to be used in cocktails  Unless further research describes developments reducing toxic compound release, studies on weakly virulent PA strains at a high load need to be avoided |
| --- | --- | --- | --- | --- | --- | --- | --- | --- | --- | --- |

| 9- Pabary, 2016 (United Kingdom) | Phage cocktail PA 24, PA 25, PA 7 (NA)  [data ureported and irretrievable] | PAO1 and PA 12B-4973 (isolated from a patient with CF) | Mice | Phage cocktail spot test on PAO1 and five CF PA strains from adult inpatient sputa (patients attending the Royal Brompton Hospital, London) | Not reported | Infected-sodium magnesium buffer-treated mice (positive) | No evidence of murine toxicity following rapid phage-induced lysis of PAO1 and CF PA strains  Evidence on the phage cocktail treatment benefits on PAO1 and CF PA infected mice. Giving phages before infecting mice with PA strains results in BAL clearance, and reduces neutrophilic inflammation | PA 12B-4973 was unusable in the delayed and prophylactic approaches owing to its virulence | No *in vitro* experiments were done to retest PA colonies recovered from mice receiving delayed or prophylactic phage doses, hence phage susceptibility and phage resistant PA strains remain unknown  Only one virulent CF strain tested in a single experimental condition owing to problems related to virulence  Lack of negative controls | Future human clinical trials testing phage cocktail efficacy in patients with CF should measure PA strain clearance in BALs  Evaluating inflammatory cell levels in mouse BAL and spleen cultures, provides reliable evidence on PA infection and dissemination |
| --- | --- | --- | --- | --- | --- | --- | --- | --- | --- | --- |

*Abbreviations: CF, cystic fibrosis; NA, data not available; PBS, phosphate-buffered saline; BAL, bronchoalveolar lavage; *in accordance with the International Committee on Taxonomy of Viruses* [*https://talk.ictvonline.org/taxonomy/*](https://talk.ictvonline.org/taxonomy/)*; **results in vitro reported in* ***Supplementary*** ***Table 3****; ***EOP, efficiency of plating = the ratio between the average PFU on target bacteria and average PFU on host bacteria -- is the standard method for testing lytic phage activity (in accordance with Mirzaei et al., 2016); ****MOI, multiplicity of infection = the ratio between the plaque-forming units (PFU) and the colony-forming units (CFU); ^†^poster presented at the European Congress of Clinical Microbiology and Infectious Diseases 2016. No published results.*
